# Mitochondrial Structure and Bioenergetics in Normal and Disease Conditions

**DOI:** 10.3390/ijms22020586

**Published:** 2021-01-08

**Authors:** Margherita Protasoni, Massimo Zeviani

**Affiliations:** 1 Mitochondrial Biology Unit, The MRC and University of Cambridge, Cambridge CB2 0XY, UK; mp802@mrc-mbu.cam.ac.uk; 2 Department of Neurosciences, University of Padova, 35128 Padova, Italy

**Keywords:** ATP production, biogenesis of the respiratory chain, mitochondrial disease, mi-tochondrial electrochemical gradient, mitochondrial potential, mitochondrial proton pumping, mitochondrial respiratory chain, oxidative phosphorylation, respiratory complex, respiratory supercomplex

## Abstract

Mitochondria are ubiquitous intracellular organelles found in almost all eukaryotes and involved in various aspects of cellular life, with a primary role in energy production. The interest in this organelle has grown stronger with the discovery of their link to various pathologies, including cancer, aging and neurodegenerative diseases. Indeed, dysfunctional mitochondria cannot provide the required energy to tissues with a high-energy demand, such as heart, brain and muscles, leading to a large spectrum of clinical phenotypes. Mitochondrial defects are at the origin of a group of clinically heterogeneous pathologies, called mitochondrial diseases, with an incidence of 1 in 5000 live births. Primary mitochondrial diseases are associated with genetic mutations both in nuclear and mitochondrial DNA (mtDNA), affecting genes involved in every aspect of the organelle function. As a consequence, it is difficult to find a common cause for mitochondrial diseases and, subsequently, to offer a precise clinical definition of the pathology. Moreover, the complexity of this condition makes it challenging to identify possible therapies or drug targets.

## 1. Mitochondria

### 1.1. Origin of Mitochondria and Mitochondrial Genome

According to current theories, mitochondria evolved from free-living bacteria and participated in the origin of eukaryotic cells through a process known as endosymbiosis [1]. The endosymbiotic hypothesis proposes that original anaerobic eukaryotic (modern views indicate endosymbiosis with a non-eukaryotic archaeon organism) cells engulfed the primitive mitochondria and established a favorable interaction (although this occurred after massive negative selection due to gene shuffling) for both the organisms. Indeed, mitochondria were able to drastically improve the cell energy production, generating adenosine triphosphate (ATP) through the respiratory chain, while the host cell offered a safe environment for bacterial proliferation [2]. 

This theory was supported by the discovery in the 1960s of mitochondrial DNA (mtDNA) and an independent mitochondrial translation system. Indeed, mitochondria contain their own genetic material, mtDNA, which maintains the typical features of bacterial DNA: it is a circular 16,569-base pairs (bp) double-stranded molecule, does not contain introns and is polycistronic [3]. In fact, apart from one non-coding region, called the displacement loop or D-loop, each gene is contiguous to the next one, albeit some are partly overlapped. Moreover, contrary to nuclear DNA, mtDNA is present in many copies in the cell, between 100 and 10,000 copies, proportionally to the energy demand of the specific tissue [3]. Finally, the mtDNA genetic code differs slightly from nuclear DNA, presenting different codons encoding for tryptophan and methionine and only two stop codons. This is true in different species, the genetic code being different, for instance, between vertebrates and other metazoans, but it is a universal code in plants; this implies that the change in the genetic code of mtDNA occurred several times during evolution and it cannot be the primary cause for the maintenance of mtDNA within the organelle.

**Figure 1:**
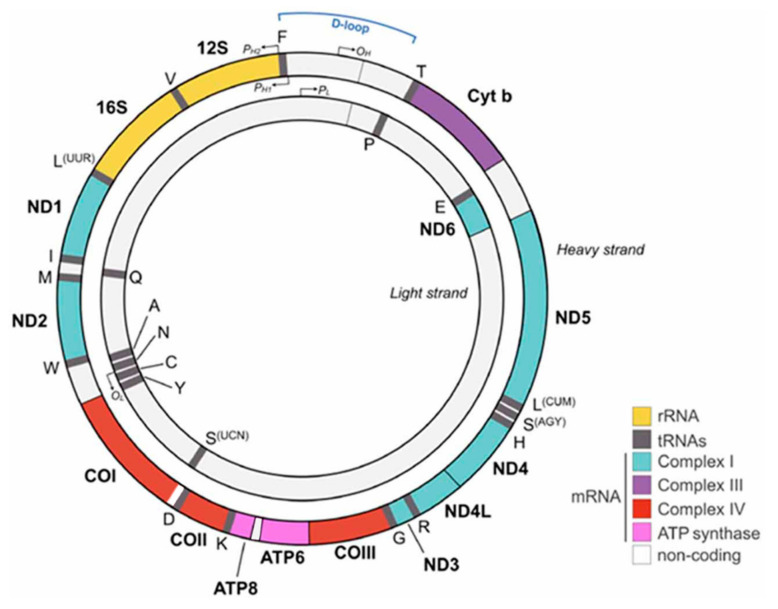
Schematic representation of mitochondrial DNA (mtDNA). Each protein-encoding gene is indicated with a colored bar and all the genes encoding for subunits of the same complex are represented with the same color. rRNAs are indicated in yellow and tRNAs in gray. Source: adapted from Hoffmann and Spengler, 2018 [6].

During evolution, most of the mitochondrial genes were lost or transferred to nuclear DNA, and today, mtDNA only contains 37 genes: 11 messenger ribonucleic acids mRNAs, translated to 13 proteins, 2 ribosomal RNAs (rRNAs, 12S and 16S) and 22 tRNAs [4]. The structure of mtDNA is represented in Figure 1.

### 1.2. Mitochondrial DNA Mutations

As for nuclear DNA mutations, alterations in mtDNA can have important pathological consequences. However, because of the differences between mitochondrial and nuclear DNA, mtDNA inheritance does not follow the canonical mendelian genetics. Firstly, in sexuate organisms, mtDNA is maternally inherited [5]; therefore, only the mother can transmit mutant mtDNA to offspring. Secondly, whilst nuclear genes are present in only two copies per cell, every cell contains multiple copies of mtDNA. These copies can all be identical in sequence, giving a condition known as homoplasmy. However, inheritance of mutated copies, replication errors, oxidative stress or inefficient DNA repair can lead to mtDNA mutations in a percentage of copies, causing heteroplasmy [3]. The proportion of mutant DNA versus the wild-type variant has a strong impact on the development and the severity of the pathological phenotypes.

### 1.3. Mitochondrial Membranes

**Figure 2:**
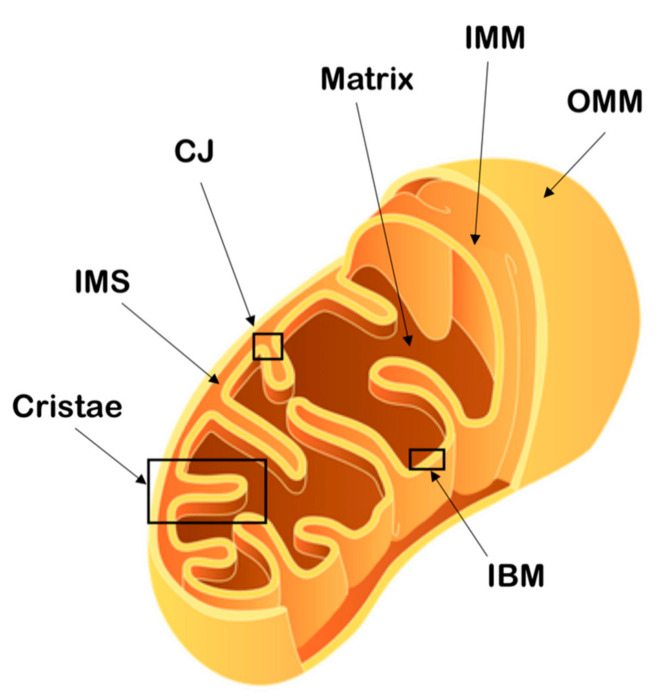
Schematic representation of mitochondrial architecture. The outer mitochondrial membrane (OMM), inner mitochondrial membrane (IMM), inner boundary membrane (IBM), cristae junctions (CJ), intermembrane space (IMS), cristae and mitochondrial matrix are indicated.

Mitochondria are surrounded by two phospholipidic membranes, the outer mitochondrial membrane (OMM) and the inner mitochondrial membrane (IMM), which divide the organelle into two spaces, the matrix and the intermembrane space (IMS) [7]. The two membranes present significant differences in lipid composition, characteristics and roles of the transmembrane proteins, permeability and shape and are the result of the endosymbiotic origin of the organelle. Indeed, the OMM is more similar in lipid composition to eukaryotic cell membranes, while the IMM resembles the cardiolipin-containing bacterial membranes [2]. The IMM is characterized by a higher protein/lipid ratio and forms highly packed invaginations in the matrix, called cristae [8]. Embedded in the cristae resides, together with many other proteins, the oxidative phosphorylation (OXPHOS) machinery and one proposed reason for the IMM folding is to increase the available surface for energy production. The part of the IMM that does not protrude in the matrix but, instead, runs parallel to the OMM is called the inner boundary membrane (IBM). Cristae and the IBM are connected via narrow tubular or slit-like structures, the cristae junctions (CJs) [9] (Figure 2).

Moreover, the OMM and IMM differ largely in their permeability. While the OMM allows the passage of ions and small molecules through voltage-dependent anion channels (VDACs) [10], only water, oxygen (O_2_) and carbon dioxide (CO_2_) can pass freely through the IMM. This selectivity allows the formation of an electrochemical gradient across the membrane, which forms the basis for ATP production, and the tight regulation of other ions concentrations, such as calcium, largely used in cell signaling [11,12]. 

### 1.4. Mitochondrial Cristae

Mitochondria are internally organized into cristae, invaginations of the IMM, which can be dynamically reorganized according to various stimuli, such as changes in energy requirements or apoptotic signals, becoming more or less compact [13]. The formation and maintenance of these structures is a complex process that requires the participation of many proteins, including the mitochondrial contact site and cristae organizing system (MICOS) and the fusion protein Optic atrophy type 1 (OPA1). The MICOS and OPA1 have been widely associated with the regulation of the cristae architecture, specifically at the cristae junction [14].

The MICOS complex was originally characterized and largely studied in yeast, where it is composed of six subunits: Mic10/MINOS1, Mic12/Aim5, Mic19/CHCHD3, Mic26/ApoO, Mic27/ApoOL and Mic60/Mitofilin [15]. In mammals, this system is more complicated and two additional subunits have been described: Mic25/CHCHD6 [16] and Mic13/QIL1 [17]. All of these subunits are transmembrane proteins, except for Mic19 and Mic25.

**Figure 3:**
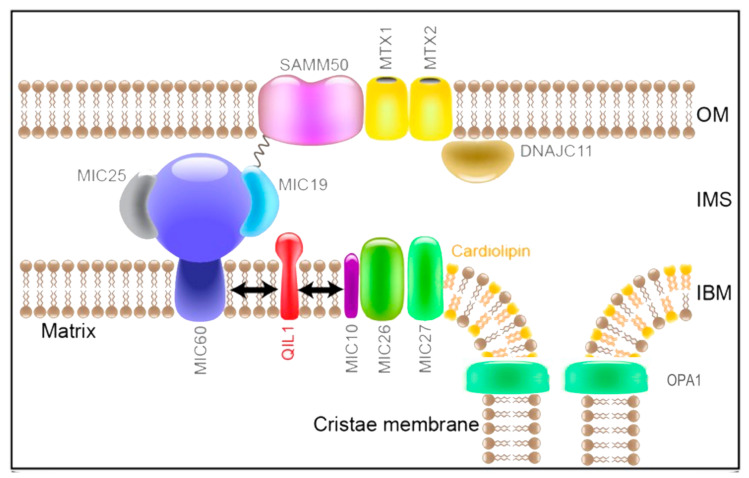
Cartoon of the mitochondrial contact site and cristae organizing system (MICOS) complex at the cristae junction. Source: adapted from Guarani et al., 2015 [17].

Mic60 and Mic10 are the core subunits of the two subcomplexes forming the MICOS architecture (Figure 3). From one side, Mic60 contacts directly with Mic19 and Mic25, while the remaining subunits assemble with Mic10 [18]. Mic10 interacts with Mic26 and Mic27 and is stabilized by QIL1 [17]. Mic10 has been shown to be able to induce membrane curvature even in the absence of the other MICOS subunits [19]. Both subcomplexes are necessary for the formation and stabilization of the cristae at the CJ. 

The Mic60/Mic19 module is also associated with the OMM. Indeed, Mic19 has been shown to form a bridge between Mic60 and Sorting Assembly Machinery 50 kDa subunit (SAM50), the outer membrane protein that regulates the import and assembly of β-barrel proteins [20], creating tight OMM and IMM contact sites. Other proteins have been specifically associated with this OMM–IMM junction such as Metaxin-1 and 2 (Mtx1/Mtx2) and DnaJ homolog subfamily C member 11 (DNAJC11) [21]. These OMM–IMM interactions create a specific environment of closed apposition of the two membranes favorable for protein import and lipid and phospholipid transport [22].

Immunoprecipitation experiments indicate that both Mic19 and Mic60 interact with OPA1. OPA1 is an IMM protein with a double role in mitochondrial dynamics and architecture: it is involved in mitochondrial fusion of the IMM and it participates in cristae remodeling [23,24]. Indeed, OPA1 has been shown to be upstream and epistatic to MIC60 and to be the sole regulator of cristae width and junction diameter and number [25]. 

Finally, the formation of the cristae rims has been proposed to be also linked to the dimerization of the ATP synthase. Indeed, Complex V (CV) dimers reside at the apex of the cristae and, when dimerization is abolished, the IMM loses the typical tubular organization of the cristae and forms onion-like structures instead, a typical feature associated with MICOS deregulation [26].

### 1.5. Protein Transport through Mitochondrial Membranes

The mechanisms of protein and ion transport through the OMM and the IMM differ in many aspects [27]. As described previously, the IMM is much less permeable than the OMM and is characterized by α-helical transport proteins such as protein translocases and other carriers for metabolites and ions. The OMM, instead, contains channel-forming proteins, such as β-barrel transmembrane hydrophilic pores, that allow the passage of precursor proteins, small hydrophilic metabolites and ions.

Human mtDNA only contains 37 genes, while it has been estimated that the mitochondrial proteome is composed of approximately 1200 proteins [28]. Consequently, most of the proteins required are encoded by nuclear genes, translated in the cytosol and transported to or into the organelle. Indeed, newly synthesized proteins carrying a mitochondrial target signal are transported to the OMM by chaperones and, according to the nature of their signal, they can be inserted in the OMM, imported in the IMS or transported to the IMM translocases [29]. At the OMM, proteins transport is ensured by the two main complexes: the Translocase of the Outer Membrane (TOM) complex and Sorting and Assembly Machinery (SAM) complex [30,31]. For insertion of proteins in the OMM, integral proteins containing one or more spanning helices are not imported, but rather inserted in the OMM via initial interaction with TOM70 (receptor subunit of TOM) and then, in yeast, with Mim1, while β-barrel proteins pass through TOM40 (pore subunit of TOM) and are then transported by chaperones to SAM. The main component of SAM is SAM50, a β-barrel protein, which interacts with the new protein, accommodates the folding and inserts it laterally into the membrane [32].

Proteins localized in the IMS can have different fates: they can undergo modifications to stabilize the protein and prevent retrograde transport, such as insertion of a heme group or oxidation of cysteine residues in order to form a disulfide bridge via the Mia40 pathway [33]. In addition, modified proteins with a specific target peptide can be directed to the IMM or the matrix via TIM23 [34]. 

Proteins that have to be inserted in the IMM are delivered by the IMS chaperones TIM9-10 to the IMM insertase/translocase complex TIM22, the main pathway for import of polytopic inner membrane proteins [35]. The import through TIM22 requires the mitochondrial membrane potential, responsible for an electrophoretic effect on the positively charged targeting sequences of these proteins, but it is not ATP-driven [36]. Finally, Oxa1, a conserved membrane protein, mediates the insertion of both nuclear and mitochondria-encoded precursors into the inner mitochondrial membrane [37].

For matrix-targeted proteins, the translocase involved in this process is TIM23 and three different forces drive the transport: the membrane potential, the increasing affinities of the precursor proteins to the components on the *trans* side of the translocase, compared with the affinities to the *cis* side, and the motor force generated upon ATP hydrolysis by the chaperone mtHsp70 and its associated subunits [38].

### 1.6. Mitochondrial Dynamics

Mitochondria are dynamic organelles that form a complex network of tube-like structures [39]. They undergo opposing fusion and fission events to generate a specific mitochondrial morphology network according to the cellular energy needs, the metabolic state of the cell or to adapt to cellular cues. Mitochondrial fusion allows the organelles to share metabolites, proteins and mtDNA, and a hyperfused mitochondrial morphology is associated with a mechanism of defense to enhance cell survival (and impede mitochondrial clearance). In contrast, while mitochondrial fragmentation is often associated with mitochondrial dysfunction and cell death, this process is also required for mitochondrial motility or segregation of damaged portions of the reticulum for degradation through a process known as mitophagy [40]. 

Mitochondrial dynamics are controlled by Guanosine Triphosphatase (GTPases) proteins belonging to the Dynamin family of proteins, where GTP hydrolysis leads to structural change, subsequently driving membranes remodeling [41]. During mitochondrial division, the constriction of the tubule and membrane scission of one mitochondrion into two separate organelles is carried out by recruitment of the cytosolic GTPase Dynamin-related/-like protein 1 (Drp1) to mitochondria–endoplasmic reticulum (ER) contact sites, via interaction with fission protein 1 (Fis1) and mitochondrial fission factor (Mff) [39]. At these sites, Drp1 oligomerizes into a ring-like structure and upon GTP hydrolysis drives mitochondrial division. Mitochondrial fusion, instead, is a two-step mechanism with the OMM localized GTPases mitofusin 1 and 2 (Mfn1 and Mfn2) ensuring OMM fusion, and the IMM GTPase OPA1 responsible for IMM fusion. Again, the shape and dynamics of mitochondria are tightly linked to their bioenergetic status. Failing mitochondria usually appear fragmented, due to a prevalence of the fission machinery, whereas mitochondria hyperfuse as a mechanism of defense against autophagy in conditions of stress, including bioenergetic stress. In addition, specific disorders of some main actors of mitochondrial fission (e.g., mutations in the dynamin-related protein, DRP, the key factor of mitochondrial fission [42,43]) or fusion (e.g., mitofusin 2, Mfn2 [44], and optic atrophy protein 1, OPA1 [45,46,47]) can be associated with OXPHOS failure and, in particular for some OPA1 mutations, with the accumulation of multiple mtDNA deleted species and multiple defects of the respiratory chain complex activities [48]. In addition to its profusion role, OPA1 acts also as a sealer of the cristae junctions and regulates the ordered distribution of the ETC complexes along the mitochondrial cristae [49,50]. Therefore, alterations in mitodynamics are, nowadays, considered part of the possible causes of OXPHOS deficiency and bioenergetic failure leading to mitochondrial disease.

Mitochondria need to move within the cell and their localization is crucial for different functions such as cell division [51]. Directed mitochondrial transport happens on microtubule filaments, typically through force-generating motor proteins, classified into three families: myosins, kinesins and dyneins [52]. Milton, syntabulin and the GTPase Miro have been identified as mitochondria-specific molecules involved in microtubule-based transport [51]. 

Mitochondrial trafficking is fundamental for a constant supply of healthy mitochondria generating ATP at the right time and place, especially in neuronal cells, where organelles have to move over long distances along the axon, from the cell body to the presynaptic terminal [53].

### 1.7. Mitochondrial Functions

Mitochondria are commonly known as the “powerhouse of the cell”, due to their role in energy production. However, in the last 30 years, mitochondria have been characterized also as a signaling organelle involved in numerous physiological functions, including calcium homeostasis, apoptosis and heme and iron-sulfur clusters synthesis. 

#### 1.7.1. Energy Production

The main source of energy in cells derives from the de-phosphorylation of an ATP molecule to an adenosine diphosphate (ADP) molecule. In order to make this process sustainable, the cell needs to use nutrients to re-generate the ATP molecules used. 

During a series of chemical reactions, indeed, a glucose molecule is gradually broken down into carbon dioxide, and its hydrogen atoms are stripped and used to combine with oxygen to form water. The first stage of this mechanism takes place in the cytosol, is anaerobic and is mitochondria-independent [54]. This pathway is known as glycolysis, and it produces only two molecules of ATP from one molecule of glucose metabolized, generating two molecules of pyruvate. 

**Figure 4:**
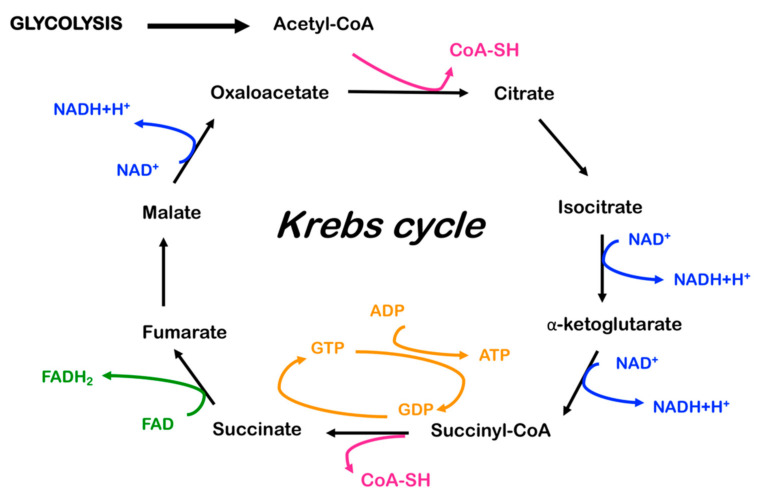
Schematic representation of the tricarboxylic acid cycle or Krebs cycle.

To optimize ATP production, glycolysis is coupled to a second pathway, known as the citric acid cycle (or tricarboxylic acid cycle or Krebs cycle, Figure 4), which takes place in mitochondria and is aerobic. This cycle is composed of nine different enzymatic reactions and each round generates three nicotinamide adenine dinucleotide (NADH) molecules, one flavine adenine dinucleotide (FADH_2_) and one guanosine triphosphate (GTP). One molecule of glucose is catabolized by glycolysis into two molecules of the ketoacid pyruvate. Pyruvate enters through the mitochondrial pyruvate carrier (MPC) into the mitochondrial matrix [55], where it is decarboxylated, oxidized and coupled with coenzyme A by pyruvate dehydrogenase, PDH, to form acetyl-CoA. Thus, the two glucose-derived acetyl-CoA molecules in the mitochondrial matrix lead to a complex catabolic cycle, the Krebs cycle, which gives rise to two molecules of CO_2_, whereas their electron equivalents (hydrogen atoms) are stripped off and used to produce, in total, six NADH, two FADH_2_ and two GTP. 

NADH and FADH_2_ molecules can then be used in a process known as oxidative phosphorylation, where the majority of the energy is finally converted to ATP. This process consists of the passage of electrons from NADH and FADH_2_ to the final acceptor, oxygen, through the electron transport chain (ETC). This process, which involves molecular oxygen as a sink for binding electrons, is called cellular respiration. Respiration is coupled with ATP production by ATP synthase or complex V of the ETC. In total, the complete oxidation of a single molecule of glucose is used by the cell to produce 30 ATP molecules [56].

Importantly, NADH and FADH_2_ can also be produced by a process known as fatty acid β-oxidation. A fatty acid is converted to fatty acyl-CoA and then to acyl carnitine, in order for it to enter the mitochondria and eventually be reconverted in intramitochondrial acyl-CoA. In the organelle, a long-chain acyl-CoA is broken down to acetyl-CoA molecules, producing one NADH and one FADH_2_ for each couple of carbons hydrolyzed from the acyl chain [57].

The proton gradient produced during respiration, similar to an accumulator, supplies energy to operate the ATP synthase (complex V) which, through dissipating it, provides the energy to condense ADP and Pi into ATP. Thus, respiration is distinct but coupled to phosphorylation, in the oxidative phosphorylation pathway. In a normal adult human being, this process leads to the daily production of approximately 70 kg of ATP, which provides the energy necessary for all exergonic reactions of the organism.

#### 1.7.2. Apoptosis

Mitochondria also play a role in the regulation of programmed cell death, called apoptosis, which is required for embryonic development and numerous physiological functions. Apoptosis leads to a controlled and programmed cell death, which can occur as a response to various damages or stressors, such as DNA damage, oxidative stress, immune reactions and absence of certain growth factors, hormones and cytokines, or as a natural part of development and aging [58]. Different apoptotic pathways exist, characterized by different triggers but with a common final execution pathway. Indeed, these different pathways lead to activation of initiator caspases (as caspase 8 and 9), which then activate executioner caspases (as caspase 3 or 7), to finally induce the degradation of cellular components. The extrinsic or death receptor pathway, which does not directly involve the mitochondria, is activated by extracellular ligands binding to death receptors on the plasma membrane and leads to the formation of the death-inducing signaling complex (DISC), which subsequently activates the initiator caspase 8 and then the executioner caspase 3 [59]. The best characterized is the mitochondrial or intrinsic pathway of apoptosis. Mitochondrial apoptosis is initiated by internal signals of stress or damage that usually lead to a bioenergetic failure and decrease ∆P and it consists in the mitochondrial outer membrane permeabilization (MOMP), regulated by the Bcl-2 family of proteins. Upon stressors, the pro-apoptotic members BAX and BAK oligomerize at the OMM [60,61], where they induce the release of pro-apoptotic proteins from the IMS into the cytosol, including cytochrome *c* [62]. Once in the cytosol, cytochrome *c* binds and activates apoptotic protease activating factor-1 (Apaf-1) as well as procaspase-9, forming a complex known as the “apoptosome”. Active caspase 9 is then able to cleave and activate caspase 3, starting the communal execution pathway [63,64]. The execution pathway leads to DNA fragmentation, degradation of cytoskeletal and nuclear proteins, cross-linking of proteins and formation of apoptotic bodies. Thus, bioenergetic failure often leads to mitochondrial apoptosis, although it may also cause autophagy of individual spent mitochondria, or even to ablation of a portion of dysfunctional portions of mitochondria that are left surviving. Therefore, apoptosis and related phenomena constitute one of the possible deleterious outcomes of mitochondrial disease. Importantly, in addition to the release of cytochrome c and formation of the apoptosome, other mitochondrial proteins can determine apoptosis through alternative mechanisms, such as the apoptosis inducing factor 1 (AIF1) [65], a redox mitochondrial membrane-bound protein that in stress conditions can be cleaved by cathepsin or other proteases, released outside mitochondria and there activate caspases independently from the formation of apoptosome, eventually leading to apoptosis. Recessive mutations of AIF1 have been reported in severe infantile syndromes associated with multiple defects of the mitochondrial respiratory chain activities [66].

#### 1.7.3. Calcium Homeostasis

Calcium is largely used in cells as a signaling molecule; therefore, its regulation is critical. Cellular organelles such as the ER and mitochondria are able to sequester and release calcium, regulating the cellular concentration of the ions. Vice versa, calcium signaling has a role in mitochondrial functionality, even if not all the molecular mechanisms involved are clear yet. What is largely accepted is that calcium in the mitochondrial matrix regulates various enzymes, such as pyruvate, isocitrate and 2-oxoglutarate dehydrogenases, modulating, as a consequence, mitochondrial respiration, and the induction of the mitochondrial permeability transition pore [67].

Calcium passes the OMM barrier through a VDAC, which is characterized by high-conductance and weak anion selectivity [68]. The passage through the IMM, instead, is more controlled and involves the mitochondrial calcium uniporter (MCU), which transports Ca^2+^ inside the matrix, and a Na^+^/Ca^2+^ exchanger, mostly expressed in excitable cells, such as muscles and brain, or an H^+^/Ca^2+^ exchanger, in other cell types, which release calcium from the matrix to the IMS [69]. 

#### 1.7.4. Heme Synthesis

Heme is an iron-containing porphyrin, essential in numerous biological processes, such as oxygen transport and storage, drug and steroid metabolism, signal transduction and microRNA processing [70]. Moreover, heme is incorporated in some subunits of the electron transport chain and it is necessary for cellular respiration [71].

The synthesis of this compound occurs both partially in the mitochondria and in the cytosol [72]. Heme is generated by the insertion of ferrous iron into the tetrapyrrole macrocycle of protoporphyrin IX [73] catalyzed by a mitochondrial matrix enzyme, called ferrochelatase. Protoporphyrin IX is produced starting from glycine and succinyl-CoA. 

Most of heme production takes place in erythroid progenitors, followed by the liver for the formation of heme-containing enzymes [73]. The synthesis pathway is conserved in these two cellular types, while its regulation differs. The heme synthesis machinery in the liver has a rapid turnover in order to respond quickly to changes in metabolic requirements, while the synthesis in developing red cells is tied to the availability of iron.

#### 1.7.5. Fe/S Clusters Synthesis

Iron-sulfur (Fe/S) clusters are prosthetic groups with a variety of biological functions. Indeed, several enzymes, such as glycosylases, helicases, primases and respiratory chain enzymes, require the incorporation of Fe/S centers for their activity [74]. Both the proteins containing these centers and the proteins that are part of the biosynthesis machinery are highly conserved in prokaryotes and eukaryotes, suggesting an important role in the origin of life [75]. These cofactors originated probably in an environment characterized by low oxygen and co-evolved when the oxygen levels started increasing in the atmosphere, leading to the adaptation of anaerobic electron transport chains for an aerobic habitat. Most Fe/S proteins contain a rhomboid [Fe_2_/S_2_], a cuboidal [Fe_3_/S_4_] or a cubane [Fe_4_/S_4_] cluster [76]. The most common protein ligand is cysteine, but also histidine, serine and arginine can form a bond [74]. 

In yeast, Fe/S clusters synthesis takes place exclusively in mitochondria, but an Fe-S cluster can also be exported from mitochondria by a specific ABC transporter in the inner mitochondrial membrane [77]. The central actor of the synthesis pathway found in eukaryotic mitochondria is known as iron-sulfur cluster assembly enzyme (ISCU), which acts as a scaffold for the initial synthesis of a [2Fe–2S] cluster. This cluster will be the basis for the formation of both mitochondrial and cytosolic Fe/S groups. The sulfide ions used in this process are obtained from cysteine side chains, thanks to the activity of enzymes called cysteine desulfurases [78], while it is not clear how iron is delivered to the ISCU, although frataxin may be involved [79]. 

## 2. The Electron Transport Chain

**Figure 5:**
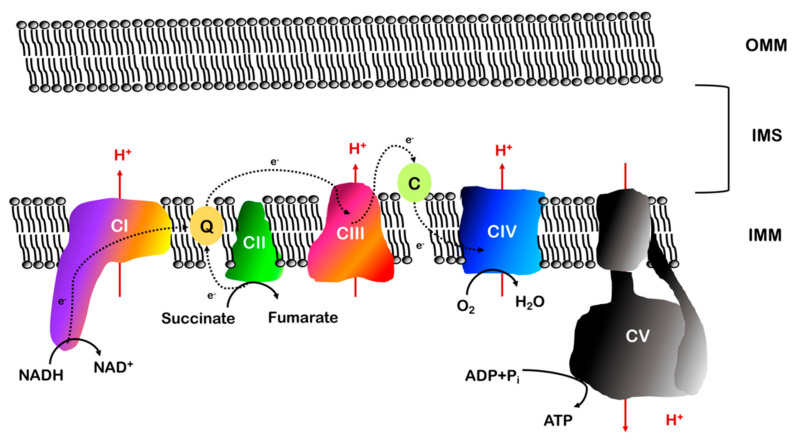
Cartoon representation of the oxidative phosphorylation (OXPHOS) machinery. NADH and FADH2 molecules generated during glycolysis and the Krebs cycle are oxidized by complex I (CI) and complex II (CII), respectively. Electrons are then passed to ubiquinone (Q), which transfers them to complex III (CIII). Here, they are transferred to cytochrome c (C) and to complex IV (CIV), where they are used to reduce O_2_ to H_2_O. Coupled to electron transfer, protons are pumped from the matrix (red arrows) to the intermembrane space (IMS) and the proton motive force generated is used by complex V (CV) to produce ATP.

The enzymatic machinery performing cellular respiration, the electron transport chain (ETC), is composed of four protein complexes embedded in the IMM and two mobile electron carriers (ubiquinone, or coenzyme Q, and cytochrome *c*) (Figure 5). Electrons are transported from electron carriers reduced during glycolysis and the Krebs cycle (NADH and FADH_2_) to coenzyme Q and cytochrome *c* and eventually transferred to O_2_, forming H_2_O. The energy liberated by this chain of redox reactions leads to the generation of an electrochemical proton gradient across the IMM, which is used by complex V or F_1_F_o_ ATP synthase to generate ATP [80,81]. 

### 2.1. Proton Gradient and Proton Motive Force

The formation of the electrochemical gradient is made possible by the nature of the phospholipidic bilayer that forms the IMM. Indeed, the membrane is impermeable to the passage of protons, which require protein transporters to cross it. These transporters are part of complexes I, III and IV and the energy necessary for the proton pumping and the generation of the electrochemical gradient derives from the transport of electrons. 

This gradient produces the proton motive force (PMF or ∆p), which can be described as a measure of the potential energy stored across the IMM. Since protons are electrically charged particles, the PMF has both chemical and electric components. The electric component corresponds to the voltage difference across the membrane and the free energy is calculated as ΔG = −F∆Ψ (F = Faraday constant; ∆Ψ = membrane potential). The chemical component, instead, has a free energy calculated as ∆G = RT ln([H^+^]_i_/[H^+^]_o_), where [H^+^]_i_ and [H^+^]_o_ refer to the proton concentrations inside and outside the IMM, respectively, *R* is the gas constant of 1.987 cal/(degree·mol), and *T* is the temperature (in degrees Kelvin). Combining these two components, the PMF is calculated as ∆p = ∆Ψ − (RT/F) * ln([H^+^]_i_/[H^+^]_o_). Under physiological conditions, the magnitude of the PMF is about −220 mV [71]. As a consequence of the difference in protons concentration, the matrix side of the inner mitochondrial membrane is negatively charged and slightly alkaline (pH = 8). 

### 2.2. Electron Transport and Oxidative Phosphorylation

**Figure 6:**
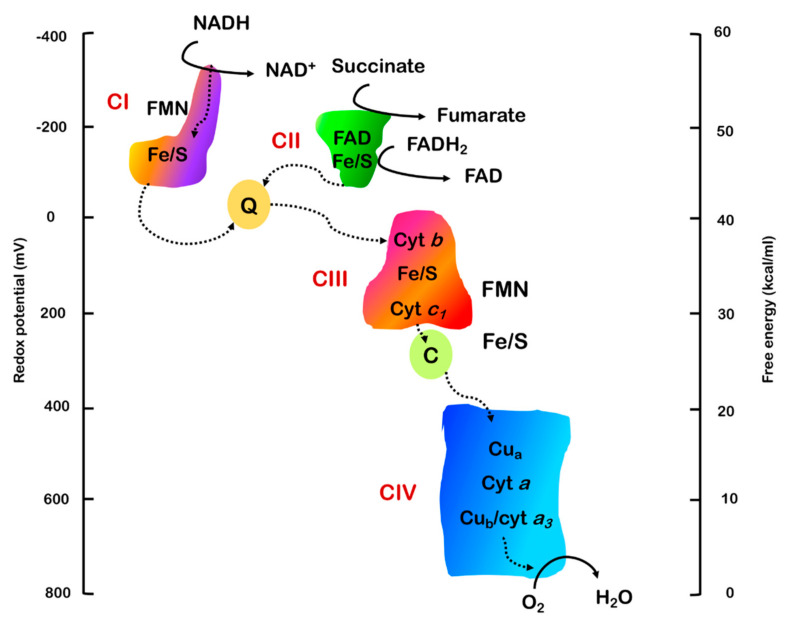
Graphic representation of electrons flow through the electron transport chain (ETC) from NADH, succinate and FADH_2_ to O_2_ (dotted arrows). The catalytic centers of the four electron transport chain complexes are represented. Electrons pass in sequence from carriers with a lower reduction potential to those with a higher potential. The energy released from the passage of electrons through the chain is coupled with the pumping of protons across the inner membrane, establishing the proton motive force (PMF). Adapted from Molecular Cell Biology, 4th edition. W. H. Freeman, New York.

The first actors in the electron transport chain are NADH and FADH_2_ (Figure 6). NAD^+^ and FAD^+^ are reduced to NADH and FADH_2_, respectively, during glycolysis or beta oxidation of fatty acids and the citric acid (Krebs) cycle. A 1:1 mixture of NADH and NAD^+^ has a redox potential of −320 mV, while the midpoint redox potential of FADH_2_ is around −220 mV. This means that both these molecules have a strong tendency to donate electrons [54]. The ΔG°’ values for these strongly exergonic reactions are −52.6 kcal/mol (NADH) and −43.4 kcal/mol (FADH_2_) [71]. 

NADH binds to complex I (CI, NADH dehydrogenase) and is oxidized to NAD^+^, donating two electrons to a flavin mononucleotide (FMN) (Figure 6), inserted in CI subunit NDUFV1. Electrons are then passed to a chain of eight iron-sulfur (Fe/S) clusters, in order to be eventually transferred to the oxidized form of coenzyme Q or ubiquinone (Q), which uptakes two protons to form ubiquinol (QH_2_). As the electrons are transferred from one redox center to the other, four protons are pumped through CI out of the matrix. Despite the numerous biochemical and structural studies on CI, a definitive model of redox-coupled proton pumping does not exist yet. Many models have been proposed: the first hypotheses suggested conformational changes in antiporter-like subunits in the P-module, allowed by the energy produced during electron transport [82,83], or transient hydration changes able to generate water-gated pathways for proton transfer between conserved ionizable residues along the membrane domain [84]. The energy necessary for the proton translocation could be provided by two processes: a two-stroke mechanism where the pumping is coupled with N_2_ (the terminal cluster in the Fe/S chain) reduction/re-oxidation, which occurs twice for every NADH oxidized, assuming the transfer of one electron at a time, or a single-stroke mechanism, where all four protons are translocated together after the reduction of coenzyme Q [85,86,87]. More recent analyses of the X-ray structure of the *Y. lipolytica* enzyme, instead, led to the hypothesis that proton pumping is linked to the coordinated conformational rearrangement of three loops in subunits ND1, NDUFS2 and ND3, triggered by the binding of negatively charged ubiquinone [88].

FADH_2_ derives from the oxidation of succinate to fumarate by complex II (CII, succinate dehydrogenase) during the Krebs cycle (and the last steps of beta oxidation), a reaction that reduces FAD^+^ to FADH_2_, a cofactor bound to the flavoprotein subunit (SDHA). Then, two electrons are transferred to the Fe/S clusters contained in SDHB, which will eventually pass them to Q (Figure 6). This process results in an increased ubiquinol pool but does not directly influence the proton gradient because CII is not a proton pump.

**Figure 7:**
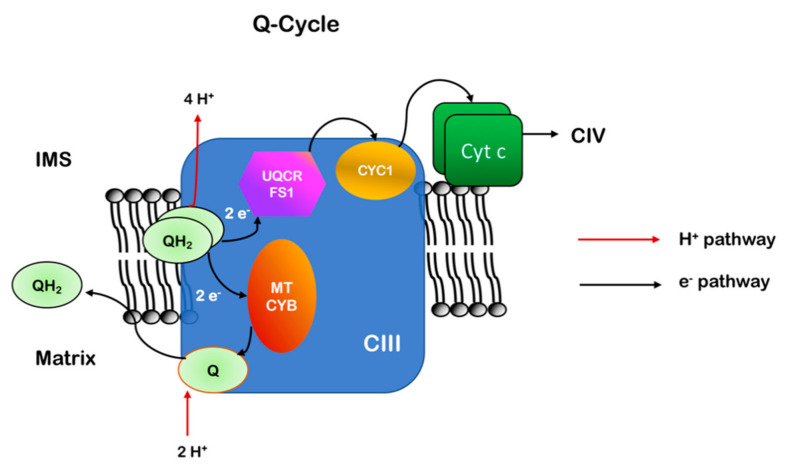
Schematic representation of the Q-cycle.

Coenzyme Q is a mobile cofactor that can interact with CI and CII and transports the electrons received to complex III (CIII, Q-cytochrome c oxidoreductase). CIII oxidizes QH_2_ to Q and passes the electrons to another soluble carrier, cytochrome *c*, during a process known as the Q-cycle (Figure 7). The Q-cycle consists of two parallel reactions, which involve the three prosthetic groups of the enzyme: the heme groups contained in cytochrome *c1* and cytochrome *b* and the 2Fe/2S cluster contained in the Rieske protein/UQCRFS1 [89]. The first reaction requires the passage of one electron from QH_2_ bound to the Q_o_ binding site to the iron/sulfur group and then to cytochrome *c1*, leading to the reduction of cytochrome *c*. Each cytochrome *c* is able to bind only one electron and, when reduced, moves from CIII to complex IV (CIV, cytochrome *c* oxidase). The second electron from QH_2_ is passed to the two heme b groups (b_L_ and b_H_) contained in cytochrome *b* and terminates on a second ubiquinone molecule bound to a different binding site of the enzyme (Q_i_ site). This ubiquinone is partially reduced to a semiquinone (Q^−^•) during the first Q-cycle and completely reduced to QH_2_ following a second catalytic cycle [90,91,92,93]. One QH_2_ molecule is then recycled and two electrons are eventually passed to two cytochrome *c* molecules. For each QH_2_ molecule that is oxidized, there is the release of two protons to the intermembrane space. QH_2_ has a redox potential around 0 mV, while CIII centers have a positive potential, allowing the passage of electrons. In order to permit the two branches of the Q-cycle, different centers of CIII must have different redox potentials, ranging between 72.5 mV for cytochrome *b*, low enough to allow the recycling of electrons through semiquinone, and 242 mV for cytochrome *c1*, which passes the electron to cytochrome *c* (251 mV) [94,95]. 

During the Q-cycle, CoQ is present in three different forms, according to its redox state: ubiquinone (Q), semiquinone (Q^−^•) and ubiquinol (QH_2_). According to the phase of the cycle and the consequent state of the reaction, CoQ molecules can bind CIII in different binding sites: Q_o_, which faces the IMS and catalyzes the oxidation of ubiquinol to ubiquinone, and Q_i_, which faces the matrix and catalyzes the reduction of ubiquinone to semiquinone and ubiquinol [96].

The last steps of oxidative phosphorylation take place in CIV (the terminal oxidase), which allows the passage of electrons from cytochrome *c* to oxygen (redox potential = 820 mV), generating water. Since cytochrome *c* carries only one electron, four molecules are oxidized in order to generate two H_2_O molecules from one molecule of O_2_. In the meantime, four substrate protons are taken from the matrix to form H_2_O and the other four protons are pumped into the IMS [97,98]. CIV contains two heme groups (cytochromes *a* and *a*_3_) and two copper atoms (Cu_A_ and Cu_B_) [99]. Electrons are transferred through the Cu_A_ center and heme *a* to the heme *a*_3_/Cu_B_ group. When both heme *a*_3_ and Cu_B_ are reduced, one O_2_ molecule is recruited to form a peroxide bridge between these two prosthetic groups. This bond is broken by the reaction with protons picked up by the mitochondrial matrix and two H_2_O molecules are formed [100]. 

### 2.3. Complex I

#### 2.3.1. Structure and Assembly

**Figure 8:**
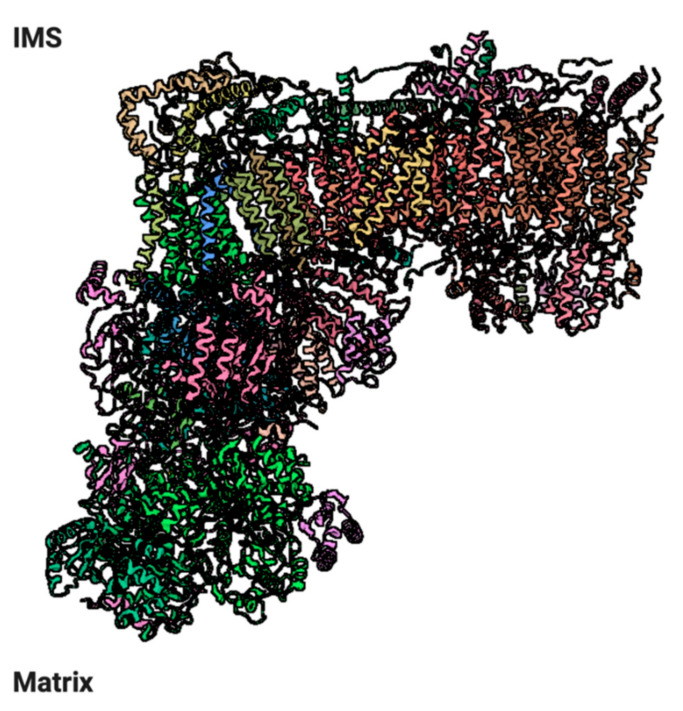
Representation of CI structure. Image has been created with BioRender.com using the structural data retrieved from PDB (5LC5).

NADH dehydrogenase (Complex I, CI) is the first step of the electron transport chain and is composed of 44 different subunits in mammals [101], organized into three structural domains: a membrane arm, or P-module, and two peripheral domains, the N and the Q modules, protruding in the mitochondrial matrix. The N module contains the FMN cofactor and is responsible for the binding and the oxidation of NADH, while the Q module contains the ubiquinone binding site. The passage of electrons between these two extremities occurs in Fe/S clusters in both the N and the Q modules. The peripheral arm is composed of nuclear-encoded proteins, 7 “core” subunits (NDUFV1, NDUFV2, NDUFS1, NDUFS2, NDUFS3, NDUFS7 and NDUFS8) and 30 accessory subunits necessary to stabilize the enzyme and to protect it from reactive oxygen species (ROS) damage [102]. The P-module, instead, is deputed to the proton pumping activity and contains seven mtDNA-encoded proteins: ND1, ND2, ND3, ND4, ND4L, ND5 and ND6. ND1 forms the reduction site for ubiquinone, while ND2, ND4 and ND5 have been found to share a similar structure to sodium and potassium antiporters and may be involved in proton pumping [103]. The mammalian CI structure is represented in Figure 8. 

Due to the large number of subunits forming CI, the assembly pathway of this enzyme is particularly complex and requires the involvement of many assembly factors. The first stage is the synthesis of the various subunits, both inside mitochondria and in the cytoplasm, coupled with the import in the organelle of the nuclear-encoded components. Most CI subunits have N-terminal mitochondrial targeting sequences (MTS), while 11 are imported into the organelle thanks to uncharacterized internal signals within the mature protein [104]. Several core subunits need further maturation and the insertion of the prosthetic groups. However, it is difficult to identify assembly factors with this role using traditional proteomic analysis, probably because of the transient and labile interaction between them and the forming enzyme [105]. The only assembly factor known to be involved in the incorporation of 4Fe/4S clusters in the peripheral arm is NUBPL, a member of the Mrp/NBP35 ATP-binding protein family [106,107]. Moreover, it is not clear if the insertion of the iron/sulfur clusters happens before or after the incorporation of the single subunit into the subcomplex. 

The second step of CI assembly is the formation of six independent modules, N, Q, ND1/P_P_-a, ND2/P_P_-b, ND4/P_D_ and ND5/P_D-b_, and the incorporation of each of them in a specific order [108]. All the known CI assembly factors are summarized in Table 1.

The ND2 module is the first detectable after inhibition of mitochondrial protein biosynthesis [109]. This subassembly binds to numerous assembly factors: ACAD9, ECSIT, TMEM126B, NDUFAF1, COA1 and the putative assembly factor TMEM186, which form the mitochondrial complex I intermediate assembly (MCIA) [110]. Moreover, TMEM186 was found to strongly interact with the newly synthesized MT-ND3, which is added to the intermediate together with MT-ND6 and MT-ND4L, forming a 385 kDa structure.

In parallel to this, an intermediate of the Q module starts forming, binding to NDUFAF3 and NDUFAF4 and generating a ~170 kDa structure. This submodule will then bind to the assembly factor TIMMDC1 and the subunits ND1, NDUFA3, NDUFA8 and NDUFA13, to yield a 283 kDa complex [109].

The ND4 module, instead, involves the subunits NDUFB1, NDUFB4, NDUFB5, NDUFB6, NDUFB10 and NDUFB11, together with the assembly factors FOXRED1, ATP5SL and TMEM70. This 230 kDa module binds initially to the N2 module and then to the ND1/Q module intermediate [109]. 

The ND5 module, which forms the distal extremity of the membrane arm, is the second to last intermediate inserted into the forming enzyme. It is composed of the subunits NDUFB2, NDUFB3, NDUFB7, NDUFB8, NDUFB9 and NDUFAB1, and it is known to bind one assembly factor: DMAC1/TMEM261 [111]. This late intermediate lacking the N module is stabilized by NDUFAF2/NDUFA12L/B17.2L. 

Finally, the N module, composed of NDUFV1, NDUFV2, NDUFS1 and NDUFA2, forming a 160 kDa assembly, is incorporated [109]. This last passage completes the assembly of the enzyme, which loses the interaction with the assembly factors and stabilizes as a ~1000 kDa complex. 

**Table 1:** CI assembly factors. Adapted from Giachin, 2016 [112], and Sanchez-Caballero et al., 2016 [105].

Assembly Factor	Function	CI Interacting Module	References
ACAD9	Binding of ND2 module	ND2/P_P_-b module	[113,114]
ECSIT	Insertion of ND2	ND2/P_P_-b module	[115]
FOXRED1	In a complex with AIFM1 and ACAD9	ND4/P_D_ module	[116,117]
ATP5SL	Binding of ND4 module	ND4/P_D_ module	[118]
TMEM70	Binding of ND4 module	ND4/P_D_ module	[119,120]
NDUFAF1	Insertion of ND2 module	N module, ND1	[121]
NDUFAF2	Binding of N module	N module	[122]
NDUFAF3	Binding of Q with P_P_-a	Q module	[123]
NDUFAF4	Binding of Q with P_P_-a	Q module	[124]
NDUFAF5	Methyltransferase activity	Not known	[125,126]
NDUFAF6	Squalene/phytoene synthase activity	Not known	[127]
NDUFAF7	Methyltransferase activity	Not known	[128,129]
NUBPL	4Fe/4S clusters insertion. Necessary for the entire enzyme stability	Supposed to interact with the developing N module and possibly Q module	[106,107,130]
TIMMDC1	Translocase of inner mitochondrial membrane domain-containing protein 1	ND1/P_P_-a	[118,131]
TMEM126B	Required for formation of the ND2 module	ND2/P_P_-b module	[132]
TMEM186	Not known	ND2/P_P_-b module	[109]
DMAC1/TMEM261	Stabilization and/or assembly of ND5	ND5/P_D-b_	[111]
COA1	CIV assembly factor, found bound to CI assembly intermediates	ND2/P_P_-b module	[109]

#### 2.3.2. Pathologies Associated with Complex I Deficiency

Mutations affecting CI stability or activity are responsible for a wide range of pathological phenotypes [133]. Missense mutations affecting the mitochondrial-encoded subunits (ND subunits) have been associated with Leber’s hereditary optic neuropathy (LHON), mitochondrial encephalomyopathy, lactic acidosis and stroke-like syndrome (MELAS) and Leigh syndrome. Many mutations in nuclear-encoded subunits have been identified in patients with CI deficiency, causing Leigh syndrome, leukoencephalopathy, leukodystrophy, encephalopathy, cardiomyopathy and other neurological defects. In addition, assembly factors and chaperones involved in CI assembly can also be at the origin of the pathogenesis of these diseases [134]. The main pathological mutations found in CI subunits or assembly factors are summarized in Table 2. 

### 2.4. Complex II

#### 2.4.1. Structure and Assembly

Succinate dehydrogenase (SDH, complex II, CII) is a ~120 kDa integral membrane complex, involved in both the TCA cycle and the ETC. Indeed, this enzyme catalyzes the oxidation of succinate to fumarate, a central step of the citric acid cycle, and reduces FAD to FADH2, which then reduces ubiquinone to ubiquinol [178]. CII is the only complex of the chain that does not pump protons across the membrane and that is entirely encoded by the nuclear DNA. 

**Figure 9:**
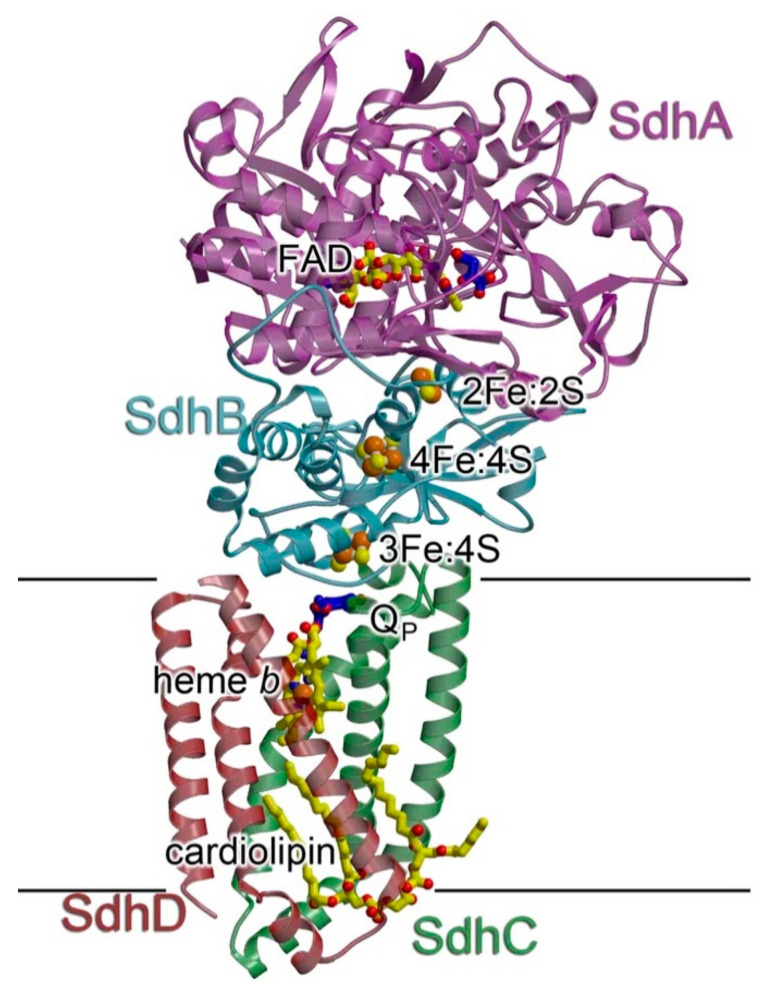
Representation of *E. coli* CII structure. The four subunits forming the complex (SDHA-D) are shown in different colors and labeled with the letters A to D. FAD, Fe-S centers, heme b and the ubiquinone binding site facing the matrix (Qp) are indicated. Source: Iverson, 2013 [181].

CII is composed of four subunits, named SDHA-D, forming two domains (Figure 9). The hydrophilic head of CII comprises SDHA and SDHB and is required for the oxidation of succinate. FAD^+^ binds to SDHA and the electrons are transferred to SDHB, containing three Fe/S clusters ([2Fe-2S], [4Fe-4S] and [3Fe-4S]) [179]. The hydrophobic membrane domain of the enzyme is composed of SDHC and SDHD and contains a heme b group and two ubiquinone binding sites [180]. 

The mature forms of SDHA and SDHB are generated independently before the complex assembly, while SDHC and SDHD are able to form an intermediate subcomplex [182]. SDHA is initially imported into the matrix as an apo-protein and the FAD cofactor is inserted thanks to the interaction with the assembly factor SDHAF2/Sdh5 [183]. Then, mature SDHA binds to SDHAF4/Sdh8, a chaperone that protects the subunit from auto-oxidation and facilitates the assembly with SDHB. Mature SDHB contains Fe/S clusters, which are inserted by SDHAF1 [184,185]. SDHB stability is then maintained by the association with an LYR motif protein recently identified in yeast, Sdh7 (SDHAF3/ACN9/LYRM10, human ortholog), which shields one or more of the prosthetic centers from oxidants [186]. Mature SDHA and SDHB are then able to assemble together and join SDHC and SDHD and insert into the membrane via a still uncharacterized mechanism. 

#### 2.4.2. Pathologies Associated with Complex II Deficiency

Patients presenting with a specific CII defect are quite rare, less than 10% of OXPHOS deficiency cases [187]. Two main phenotypes can originate from mutations in CII subunits or assembly factors. Mutations in SDHAF1 and SDHA lead to encephalomyopathy and leukoencephalopathy [184,188], while variants in SDHA, SDHB, SDHC, SDHD and SDHAF2 are responsible for hereditary paraganglioma and pheochromocytomas, rare neuroendocrine tumors [183,189,190,191,192]. Moreover, other genes involved in FAD and Fe/S cluster synthesis can impair CII activity and stability [193]. The main pathological mutations found in CII subunits or assembly factors are summarized in Table 3.

### 2.5. Complex III

#### 2.5.1. Structure and Subunits

**Figure 10:**
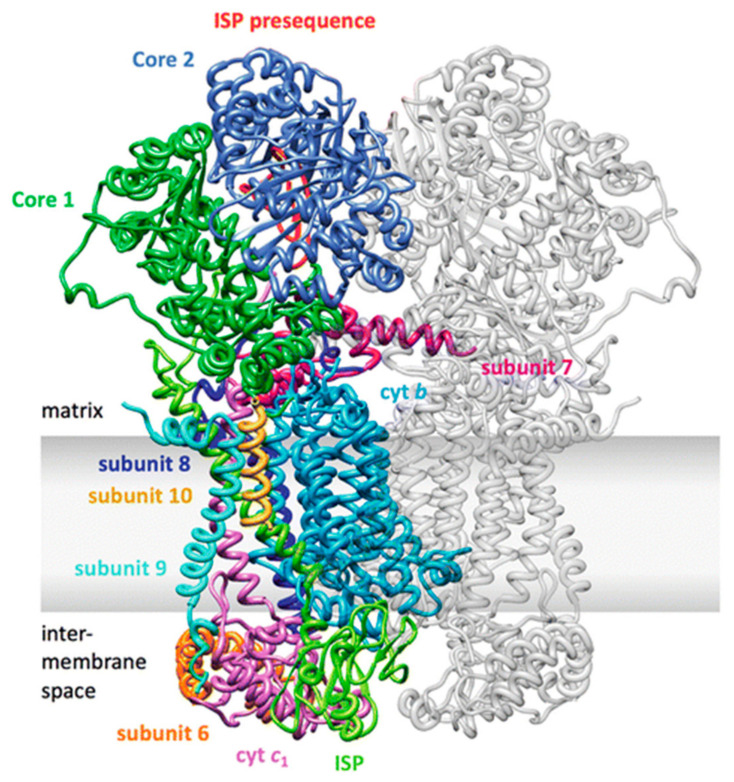
Representation of bovine CIII structure. CIII is shown as a dimer, the only form in which it is found in cells. All the 10 subunits are represented with a different color in one monomer. Source: Sousa et al., 2018 [207].

The ubiquinol:cytochrome *c* oxidoreductase (cytochrome *bc*_1_, complex III, CIII) is the central element of the respiratory chain. In yeast, it is formed of 10 different subunits, while in mammals, an additional subunit was identified, corresponding to the mitochondrial targeting sequence of the Rieske protein/UQCRFS1, which remains anchored to the complex after the proteolytic cleavage [200]. However, recent studies proposed that the latter is not a stoichiometric subunit and that the N-terminal UQCRFS1 peptide needs to be eliminated in order to maintain the functionality of CIII [201,202]. All CIII subunits are encoded by nuclear DNA except cytochrome *b* (MTCYB), which is mitochondrial-encoded [92,203]. CIII is always dimeric and high-resolution crystal structures of the bovine, chicken and yeast *bc*_1_ complexes have been resolved [91,204,205,206] (Figure 10).

Both in yeast and mammals, CIII contains three protein subunits with redox prosthetic groups: cytochrome *b*, which contains both the high-potential *b*_H_ (*b*562) and the lower-potential *b*_L_ (*b*565) heme centers, cytochrome *c1* (CYC1), containing the *c*-type heme *c*_1_, and the Rieske iron-sulfur protein (Rip1 in yeast, UQCRFS1 protein in mammals) with a 2Fe–2S cluster [208]. The di-heme cytochrome *b* polypeptide forms eight transmembrane helices and contains two histidine residues in each of the second (helix B) and fourth (helix D), forming the binding site for quinone [209]. The low-potential heme *b*_L_ is located on the intermembrane space side of the IMM, while the high-potential heme *b*_H_ is positioned in a cavity accessible from the matrix, where it can receive electrons from *b*_L_ and pass them to the Qi site, where it reduces one bound ubiquinone to semiquinone [208]. CYC1 has a wedge-like structure containing the heme group and is anchored in the membrane through a C-terminal transmembrane anchor next to helix E of cytochrome *b* [93]. The heme group binds a CXXCH domain, highly conserved in c-type cytochromes. UQCRFS1 contacts MTCYB on one of the two CIII heterodecamers with its N-terminal transmembrane domain, where it receives one electron and undergoes a conformational change that makes it reach CYC1 on the other one [210]. The maturation of UQCRFS1 has been studied in detail in simpler organisms, such as *Neurospora crassa* and *Saccharomyces cerevisiae* [211]. The newly synthesized protein undergoes two post-translational modifications: the cleavage of a targeting pre-sequence and the insertion of the iron-sulfur cluster into the mitochondrial matrix. Initially, the MTS is cleaved by a mitochondrial matrix protease (MPP), and finally, an extra eight-amino acid-long sequence is removed by a mitochondrial intermediate protease (MIP). Contrary to yeast and birds, mammalian UQCRFS1 maturation generates a 78-amino acid-long fragment, which remains temporally bound to CIII as an eleventh subunit, Subunit 9 (Su9) [200]. This additional subunit localizes between the two core subunits UQCRC1 and UQCRC2 and it has been proposed that these two are responsible for the cleavage of UQCRFS1, due to the conservation of their MPP function [212,213,214]. 

The remaining subunits are accessory and their function is mainly to support and stabilize the complex [215,216]. 

#### 2.5.2. Assembly

The CIII assembly pathway has been studied in depth in *S. cerevisiae* [217,218,219,220,221], while the human CIII assembly model has been initially constructed by homology, as some of the steps have been shown to be analogous to yeast [222], and recently updated following our study on CIII-deficient cybrids [223]. 

The first step of CIII assembly, both in yeast and in mammals, is the synthesis and the insertion into the IMM of cytochrome *b*. Yeast cytochrome *b* contains introns and requires processing [224], while mammalian *MTCYB* is transcribed as a polycistronic segment. MtDNA is organized so that mRNAs coding for proteins are divided by tRNAs, which assume a specific secondary structure, and function as punctuation marks between the genes. tRNAs are then cleaved by mitochondrial RNase P at the 5′ ends and by RNase Z at the 3′ ends, and mRNAs are then translated [225]. Moreover, studies in mice suggested the involvement of PTCD2 (pentatricopeptide repeat domain protein 2) in processing the pre-processed *ND5*-*CYTB* RNA transcript [226]. 

The transcription and translation of cytochrome *b* must be coordinated with the synthesis of nuclear-encoded proteins. This mitochondrial–nuclear communication is possible thanks to a group of proteins called translational activators. These nuclear-encoded factors regulate the expression of mitochondrial genes and their own expression in relation to the OXPHOS activity, in order to limit the accumulation of unused subunits, which can have toxic effects on the organelle. In yeast, four translational activators of COB have been identified: Cbp1, Cbs1, Cbs2 and the complex Cbp3/Cbp6 [220]. These factors interact with mitochondrial ribosomes and the mitochondrial organization of gene expression (MIOREX) complex [227]. The primary role of Cbp1 is to protect *COB* mRNAs and to transfer them to the translational apparatus [228]. Cbs1 and Cbs2 have been found associated in the same high-molecular weight complex with mitochondrial ribosomes, but they might also form other subcomplexes including those with different activator proteins, such as COX-specific activators [229,230]. Recent studies on Cbs1 determined that this protein binds to a segment of the 5′ UTR of *COB* mRNA, sequestering it and repressing the translation. Cbs1 is then replaced by the complex Cbp3/Cbp6 liberated during assembly, which activates the translation [231]. These proteins, necessary for the stability of COB mRNA and its translation, do not have orthologs in mammals [220]. 

The Cbp3-Cbp6 complex, instead, plays a role in the second phase of translation, binding the nascent polypeptide exiting the ribosome, and it does not leave the protein until the incorporation of the b_L_ heme group [232,233]. This complex has orthologs in mammals named ubiquinol-cytochrome *c* reductase complex assembly factors 1 and 2 (UQCC1 and UQCC2), with the same function [234]. Cbp3 interacts directly with Cbp4 (human ortholog: UQCC3), an assembly factor anchored in the IMM and protruding into the intermembrane space. Cbp4 is not necessary for complete translation and release from ribosomes of cytochrome *b*, but it has a role in the stabilization of the semihemylated intermediate that contains b_L_ [232,233]. Together, Cbp3–Cbp6, Cbp4 and cytochrome *b* compose intermediate I. In yeast, the now mature cytochrome *b* forms a subcomplex with the subunits Qcr7 and Qcr8, called intermediate II. Deletion of any of the genes encoding cytochrome *b*, Qcr7 or Qcr8 leads to the almost complete loss of the other two subunits and Qcr6, while the other subunits are only partially reduced [235]. Similarly, the mammalian orthologs UQCRB and UQCRQ are incorporated in the early stages of CIII assembly, provoking the release of the UQCC1-UQCC2 complex. 

The following steps of CIII assembly, instead, have been proposed to differ between yeasts and mammals. The order of incorporation in *S. cerevisiae* was determined by generating yeast mutants for single CIII subunits and studying the stability of the remaining components of the complex [217,218,219]. The third intermediate step involves the insertion of four subunits: Qcr6 (UQCRH in humans), the two large structural core subunits Cor1 and Cor2 (UQCRC1 and UQCRC2), and cytochrome *c1* (Cyt1). At this stage, dimerization occurs by joining assembly intermediate II and the Cor1/2 modules [236]. Interestingly, Cor1, Cor2 and cytochrome *c1* were found associated in a subassembly module, even if the contacts between the core proteins and the catalytic subunits in the fully assembled enzyme are minimal [217]. Surprisingly, the complex Cor1/Cor2 was detected in various subcomplexes in two-dimensional electrophoresis. This behavior might be due to the association of these subunits with other proteins or ETC complexes in the IMM or to the formation of Cor1/Cor2 aggregates. However, these subassemblies were detectable only in mutant strains and disappeared when the complex was assembled correctly; therefore, they might not represent a physiological intermediate. The last assembly factor that might have a role in the early or intermediate phases of CIII assembly is Bca1, an inner membrane protein found only in fungi [237]. However, its function is not clear yet. 

**Figure 11:**
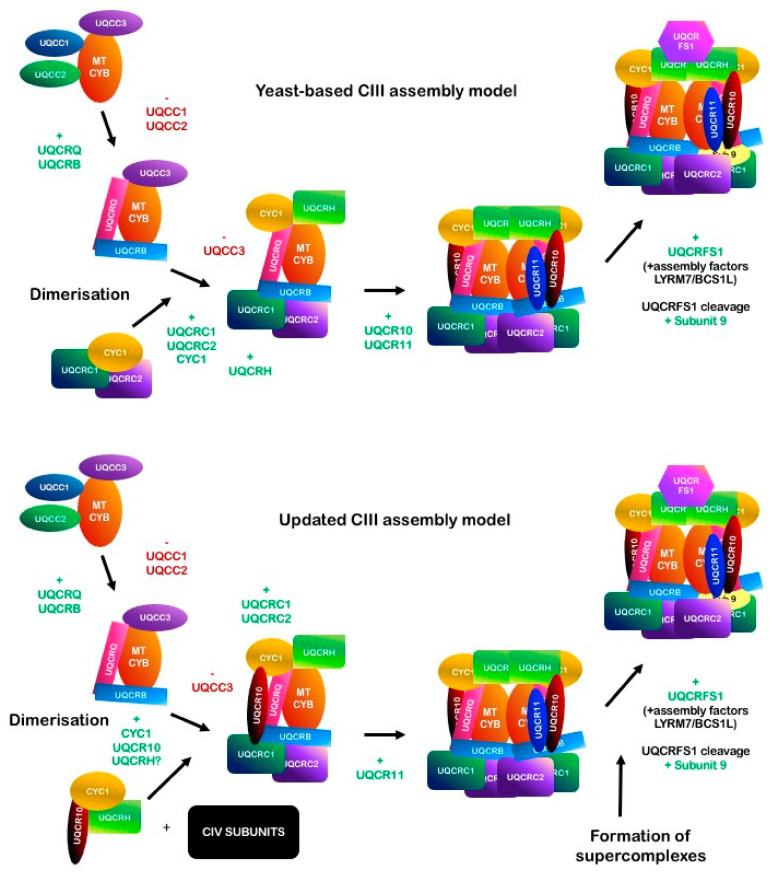
Schematic representation of human CIII assembly model based on the homology with the available data for *S. cerevisiae* [217,218,219,221,232,233,236,250,254,257], and of our updated model [220].

Taking advantage of CIII-deficient transmitochondrial cybrids carrying a mutation in *MTCYB*, however, we recently highlighted two important differences between these steps of CIII biogenesis in yeasts and in humans [223]. Firstly, we observed the formation of subassemblies containing CYC1, UQCR10 and potentially UQCRH, while our data did not suggest any interaction of CYC1 with the core subunits UQCRC1 and UQCRC2. Secondly, we identified CIV subunits, mainly belonging to the MTCO2 module, consistently interacting with this intermediate in the CIII-mutant cells. These results suggest that CIII might use CIV or CIV modules as a structural scaffold in a physiological context, or sequester CIV-specific subunits or intermediates when supercomplex formation is impaired, as a control mechanism to inhibit the complete biogenesis of the enzyme. The yeasts-based and the updated CIII assembly models are shown in Figure 11.

A requirement for the generation of intermediate III is the synthesis, the import and the maturation of Cyt1. Cyt1 contains a single heme center and is anchored to the IMM via a single transmembrane segment near its C-terminus [91]. The precursor of this subunit is translated in the cytosol and transported through TOM and TIM complexes into the mitochondria. The cytochrome *c*_1_ precursor protein contains an N-terminal cleavable bipartite pre-sequence [238]. The first of the two independent sequences, a strongly basic region of 35 amino acids, is a mitochondrial targeting signal and it is proteolytically removed by MPP in the matrix. The second sequence is a hydrophobic sorting sequence, which targets Cyt1 to the IMM. However, two models have been proposed to explain this process. The first one proposes that the whole protein, and therefore both the targeting sequences, reach the mitochondrial matrix and that only later is the second sequence re-located into the membrane, allowing the proteolytic cleavages [239]. Instead, in the second model, only the first segment reaches the mitochondrial matrix, while the second internal hydrophobic sequence remains anchored in the membrane, stopping the import. In the matrix, the positive-charged mitochondrial targeting sequence is cleaved by MPP. At the same time, the C-terminal alpha-helix gets inserted into the membrane and the heme center is attached to the protein. The hemylation is mediated by holocytochrome *c*_1_ synthetase (Cyt2 or HCCS1 in mammals) [240]. This modification provokes a conformational change that allows the exposure and the cleavage of the second targeting sequence by Imp2 (inner membrane peptidase 2), leaving the N-terminus of the mature Cyt1 soluble in the intermembrane space [241]. 

The late assembly stages involve the incorporation of Qcr9 (mammalian UQCR10) in yeasts, and Qcr10 (UQCR11) and Rip1 (UQCRFS1) both in yeasts and mammals. Firstly, Qcr9 is inserted [242]. This small (7.3 kDa) accessory subunit is necessary for the functionality of the complex and its deletion results in the formation of a nearly inactive enzyme. Indeed, it has been observed that, lacking Qcr9, the conformation of Rip1 is altered and the Fe/S cluster is not incorporated correctly [243]. The last two proteins to be inserted are Qcr10 and Rip1. Qcr10 is an 8.5 kDa supernumerary subunit incorporated before Rip1 and required for its stabilization. However, it is not clear yet how and when it is recruited [244]. Many studies, instead, have been published about the maturation and insertion of the Rieske protein both in yeast and mammals. 

Prior to the insertion, Rip1 is imported into mitochondria and receives its 2Fe/2S center, likely by the resident iron-sulfur cluster (ISC) system. In yeast, the import is followed by two proteolytic steps that eliminate the N-terminal MTS. The precursor form is first processed by a matrix MPP protease into an intermediate form [245]. The second cleavage generates the mature form of the protein and is catalyzed by the mitochondrial intermediate peptidase (MIP). At this point, Rip1 is transported back across the IMM into the intermembrane space, where it is assembled in the complex. In mammals, however, the UQCRFS1 N-terminal import signal is cleaved in a single step when the protein is already incorporated in the complex and the cleaved segment remains attached to the enzyme [200]. 

Two assembly factors are necessary for the Rieske protein assembly in both mammals and yeast: Bcs1 (BCS1L in mammals) and Mzm1 (LYRM7). Bcs1 is a 456 amino acid protein formed by three different domains: a positively charged 126 amino acid N-terminal targeting signal [246], a central Bcs1p-specific domain and a highly conserved C-terminal AAA-ATPase domain [247]. It has been initially proposed that Bcs1p might have a role in Fe/S cluster insertion, act as a chaperone [248] or bind to the partially formed CIII in an ATP-dependent manner, keeping it in a state that allows the incorporation of the Rieske protein [249]. The most recent theory is that Bcs1 is responsible for the export of the Rieske Fe/S domain from the matrix into the intermembrane space [250]. Bcs1, indeed, is able to recognize the correctly folded Rieske protein and act as a protein translocase. This model has been confirmed by the determination of the cryogenic electron microscopy (cryo-EM) structure of Bcs1 in yeast [251] and mice [252], which suggested an airlock-like mechanism for Rip1/UQCRFS1 translocation. Bcs1, indeed, seems to form two large aqueous vestibules, a bigger one on the matrix side and a smaller one in the inner membrane, through which the Rieske protein is transported. 

Mzm1 is a 14 kDa protein located in the mitochondrial matrix. It was initially thought to be involved in the modulation of the zinc pool and to this function it owes its name (mitochondrial zinc maintenance 1) [253]. However, in addition to the reduced zinc pool, cells lacking Mzm1 have a defect in CIII due to faulty insertion of Rip1 [254]. Its role is to stabilize Rip1 in the matrix before the translocation to the IMM. The same function is shared by the human ortholog, LYRM7 or MZM1L [255]. 

Finally, the third factor necessary for UQCRFS1 metabolism, which does not have a yeast ortholog, is TTC19 [256]. TTC19 binds to CIII after the incorporation of UQCRFS1 and is involved in the clearance of UQCRFS1 fragments, a process that is necessary to keep the complex in a functionally competent state [201]. A complete list of CIII subunits is indicated in Table 4 and the latest model of the CIII biogenesis pathway is represented in Figure 11.

**Table 4:** List of CIII subunits and factors involved in CIII assembly, both in *S. cerevisiae* and humans.

*S. cerevisiae*		*Homo sapiens*			
Gene	Protein	Gene	Protein		Reference (ID Yeast)
Complex III subunits					
COR1	Cor1	*UQCRC1*	UQCRC1		[258]
COR2	Cor2	*UQCRC2*	UQCRC2		[259]
COB	Cyt*b*	*MT-CYB*	Cytochrome *b*		[260]
CYT1	Cyt*c1*	*CYC1*	CYC1		[238]
RIP1	Rip1	*UQCRFS1*	UQCRFS1		[261]
QCR6	Qcr6	*UQCRH*	UQCRH		[262]
QCR7	Qcr7	*UQCRB*	UQCRB		[263]
QCR8	Qcr8	*UQCRQ*	UQCRQ		[264]
QCR9	Qcr9	*UQCR10*	UQCR10		[242]
QCR10	Qcr10	*UQCR11*	UQCR11		[244]
-	-	*UQCRFS1*	UQCRFS1		-
Translation factors				**Function**	**Reference**
CBP1	Cbp1	-	-	Translational activator of COB mRNA	[265]
CBS1	Cbs1	-	-	Translational activator of COB mRNA	[266]
CBS2	Cbs2	-	-	Translational activator of COB mRNA	[266]
CBP3	Cbp3	*UQCC1*	UQCC1	Translational activator of COB	[267]
CBP6	Cbp6	*UQCC2*	UQCC2	Translational activator of COB	[268]
Assembly factors					
CBP3	Cbp3	*UQCC1*	UQCC1	Cytochrome *b* assembly factor	[267]
CBP6	Cbp6	*UQCC2*	UQCC2	Cytochrome *b* assembly factor	[268]
CBP4	Cbp4	*UQCC3*	UQCC3	Cytochrome *b* assembly factor	[269,270]
FMP25	Bca1	-	-	Early/intermediate stages assembly factor in fungi	[237]
CYT2	Cyt2	*VPS53*	HCCS1	Heme lyase (Cytochrome *c1*)	[240]
CYC2	Cyc2	-	-	Cytochrome *c1* and cytochrome *c* assembly factor	[271]
BCS1	Bcs1	*BCS1L*	BCS1L	AAA-ATPase involved in Rieske protein incorporation	[248,249,250,272]
MZM1	Mzm1	*LYRM7*	LYRM7	Matrix protein involved in Rieske protein incorporation	[253,255,257]
-	-	*TTC19*	TTC19	Rieske protein metabolism	[256]

#### 2.5.3. Pathologies Associated with Complex III Deficiency

Pathologies due to deficiencies in CIII activity are relatively infrequent and most of them derive from mutations in *MTCYB*, the only mtDNA-encoded subunit. Mutations in this protein are generally associated with myopathy and exercise intolerance [193]. Defects in nuclear-encoded subunits are rarer, but a handful of mutations have been found in several patients (Table 5). The majority of the pathological variants associated with mitochondrial CIII deficiency of nuclear origin are found in *BCS1L* [273]. The genes found mutated in patients with CIII deficiency and the relative clinical phenotypes are summarized in Table 5. 

### 2.6. Complex IV

#### 2.6.1. Structure and Subunits

**Figure 12:**
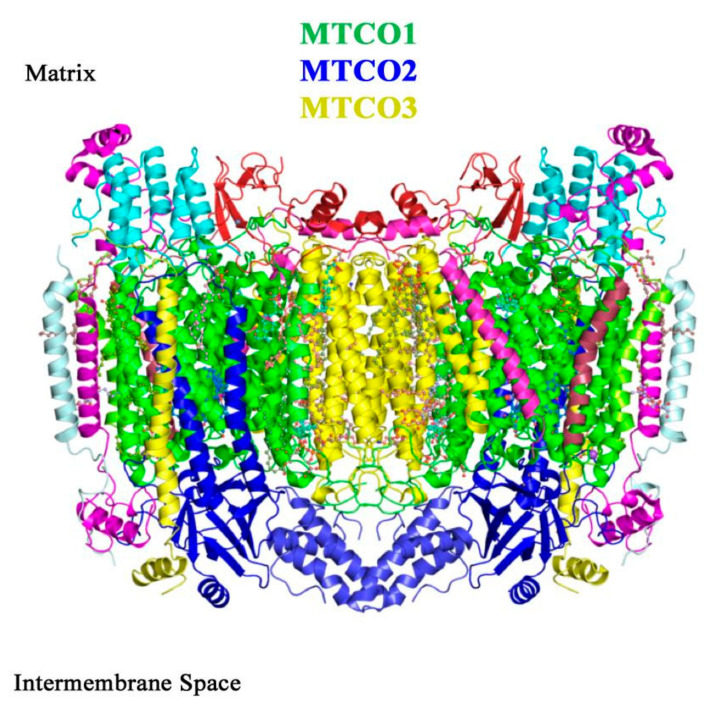
Representation of bovine CIV dimeric structure, obtained from X-ray crystallography (SFX). The functional core of the complex is composed of the mitochondrial-encoded subunits MTCO1 (green), MTCO2 (dark blue) and MTCO3 (yellow). Source: adapted from Ishigami et al., 2017 [303].

Cytochrome *c* oxidase (COX, complex IV, CIV) is the terminal step of the ETC. The enzyme has a molecular mass of about 200 kDa and in mammals it is composed of 13 subunits, 10 nuclear-encoded and 3 encoded by the mtDNA (MTCO1, MTCO2 and MTCO3), which form the functional core of the complex [296] (Figure 12). However, recently a 14th subunit, NDUFA4, previously attributed to CI, has been described [297,298] and was found to be incorporated in the structure of monomeric human CIV [299].

MTCO1 contains three prosthetic groups: cytochrome *a*_3_ and Cu_B_, which form the bi-nuclear center that binds oxygen, and cytochrome *a*. MTCO2 incorporates the Cu_A_ center [300] and MTCO3 does not have catalytic activity. The remaining subunits (COX4, 5A, 5B, 6A, 6B, 6C, 7A, 7B, 7C, 8A) are thought to have a structural role in the stabilization of the complex. Interestingly, CIV is the only ETC complex that evolved tissue-, developmental- and species-specific isoforms for COX subunits 4, 6A, 6B, 7A, 7B and 8A [301], probably in order to regulate ATP and energy production in different conditions [302]. 

#### 2.6.2. Assembly

CIV assembly is now understood as a modular process. MTCO1 was classically considered the “seed” around which the rest of the complex assembles [304,305]. However, recent evidence indicates that the first CIV subassembly is formed by the association between two nuclear-encoded subunits, COX4I1 and COX5A [306]. This module contains also HIGD1A, a protein initially proposed to be involved in the regulation of CIV activity during hypoxia [307]. 

In parallel, the MTCO1 module, also known as “MITRAC” (MItochondrial TRanslation Regulation Assembly intermediate of Cytochrome *c* oxidase) [308,309], is formed, composed of the CIV subunit and a series of assembly factors necessary for its maturation and stabilization. The first level of regulation of this module is translational, with the activity of the mitochondrial RNA-binding protein LRPPRC [310] and the translational activator TACO1 [311]. After translation, the newly synthesized protein has to be inserted in the IMM. The first factors binding MTCO1 are COX14/C12ORF62 and COA3/CCDC56/MITRAC12 [308,312,313,314]. These two chaperones assist MTCO1 during and after its insertion in the membrane and avoid the aggregation of MTCO1 subunits. The transient complex MTCO1/COX14/COA3 is stabilized by another assembly factor, CMC1 [315]. 

At this point of the pathway, MTCO1 needs the incorporation of the three prosthetic groups. Heme *a* biosynthesis is carried out by COX10 and COX15 catalyzing the conversion of heme *b* to heme *o*, and then heme *o* to heme *a* [316,317]. On the other hand, the assembly factor SURF1 has been proposed to participate in its delivery [305]. In addition, PET117 might also have a role in this process because it was found interacting with COX15 in yeast, but its involvement still has to be shown in mammals [318]. Cu_B_ incorporation is mediated by the metallochaperone COX11 [319], which is maintained in the correct redox state by COX19 [320]. The coppers are donated by COX17 [321]. 

MTCO2 requires binding with COX18 in order to be inserted in the IMM [322] and with COX20/FAM36A and TMEM177 for stabilization [323,324]. Secondly, the Cu_A_ center must be inserted in MTCO2. This process is regulated by the copper-binding proteins COX17, SCO1 and SCO2 [325,326,327], together with COA6 [328,329] and COX16 [330,331]. The MTCO2 module (MTCO2 + COX5B + COX6C + COX7C + COX8A and, most probably, COX7B) is incorporated in intermediate steps of the assembly process by joining the COX4I1-COX5A and the MTCO1 modules, forming the “S3” intermediary. This intermediary binds three assembly factors, PET100 [332], PET117 [318] and MR-1S [306]. MR-1S is a vertebrate-specific chaperone that interacts with the highly conserved factors PET100 and PET117. An additional assembly factor, APOPT1 or COA8, was proposed to have a role in the intermediate steps of CIV assembly in mouse and human mitochondria (Signes et al., 2019). Finally, the MTCO3 module (MTCO3 + COX6A1 + COX6B1 + COX7A2) is incorporated [306], followed by NDUFA4 initially described as a CI subunit and later assigned to CIV [305]. All known CIV assembly factors are summarized in Table 6.

#### 2.6.3. Pathologies Associated with Complex IV Deficiency

After CI-related pathologies, defects in CIV are the most common OXPHOS defects associated with mitochondrial disease [193]. The most frequent clinical phenotypes associated with CIV deficiencies are myopathy, affecting the skeletal muscles, and systemic pathologies, such as Leigh’s disease and multiorgan failure (https://rarediseases.org/rare-diseases/cytochrome-c-oxidase-deficiency/). As in most of the mitochondrial diseases, symptoms can be very heterogeneous and the severity of the pathology can vary greatly [354]. While most of the pathological mutations found in patients are associated with assembly factors of the enzyme or with mitochondrial tRNAs (mutations in tRNA^Lys^, tRNA^Ala^, mt-tRNA^Phe^, tRNA^Leu^, tRNA^Trp^, tRNA^Asp^ and tRNA^Glu^ were found associated with COX deficiency [354], only few cases of mutations in CIV structural subunits have been reported. This observation suggests that mutations in CIV subunits might be incompatible with life. The genes found mutated in patients with CIV deficiency and the relative clinical phenotypes are summarized in Table 7. 

### 2.7. Complex V

#### 2.7.1. Structure and Assembly

**Figure 13:**
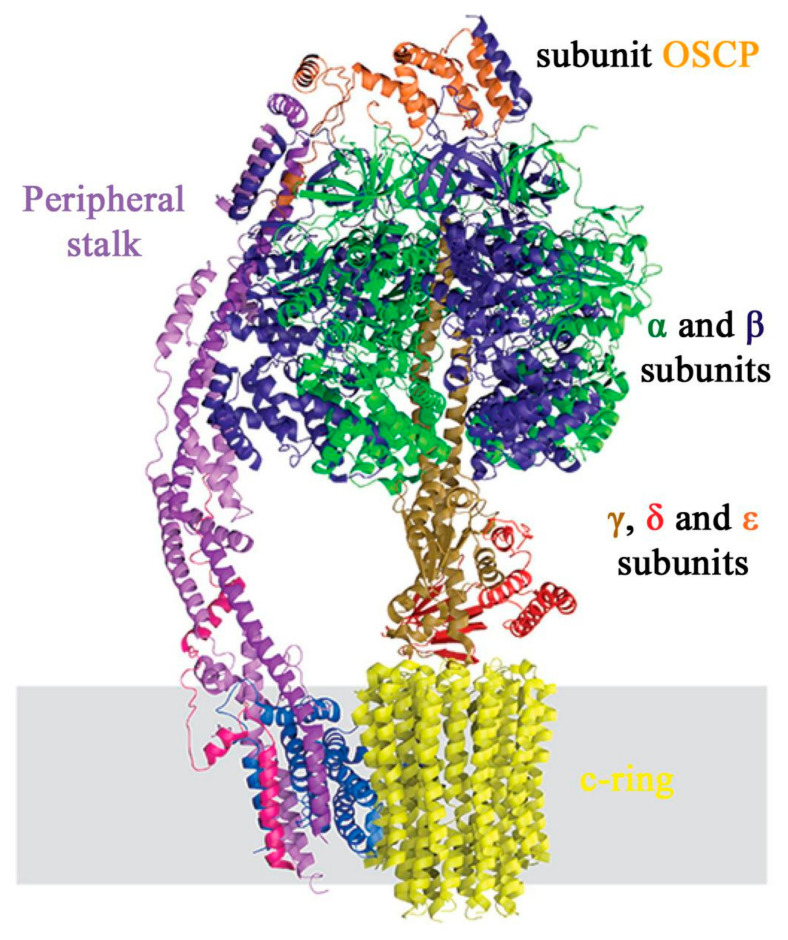
Representation of *S. cerevisiae* CV structure. The main subunits of the enzyme are indicated and shown in different colors. Source: adapted from Kuhlbrandt, 2019 [394].

ATP synthase (Complex V, CV) is the enzyme that catalyzes the synthesis of ATP from ADP and phosphate. It is composed of two distinct domains: the F_1_ domain, which faces the mitochondrial matrix, and the F_o_ domain, located in the IMM [390,391]. The human CV is composed of 29 proteins of 18 kinds, only two of which are encoded by the mtDNA (ATP6 and ATP8) [392]. A schematic representation of the main domains of CV is shown in Figure 13. The F_1_ domain is composed of three copies of subunits α and β, and one copy of subunits γ, δ and ε. γ, δ and ε subunits form the central stalk of the complex, while α and β are the subunits that physically interact with the ADP and ATP molecules [390]. F_o_, instead, is composed of a ring of c subunits and one copy each of subunits a, b, d, F_6_ and the oligomycin sensitivity-conferring protein (OSCP). The c-ring stoichiometry is not constant, but can vary, ranging from 8 copies in vertebrates to 15 in photosynthetic organisms [393]. 

Each c subunit is able to bind one proton in the IMS, which interacts with a conserved carboxylate group from a glutamate or aspartate side chain. The protonation of these subunits provokes the rotation of the c-ring, until the final dissociation of the proton at the matrix side favored by the positive charge on a conserved arginine residue (A210) of subunit a [56]. The c-ring is structurally linked to the γ and ϵ subunits and its rotation provokes the turn of these subunits inside the α_3_β_3_ hexamer unit of F_1_. On the external side, the α_3_β_3_ hexamer is prevented from rotating by the peripheral stalk formed by the two b chains and the d subunit. The result of the proton transport, therefore, is first the rotation of the c-ring, followed by the rotation of the γ subunit, and the consequent synthesis of ATP through the binding change mechanism of α_3_β_3_. The binding change mechanism is based on the fact that the interactions between the γ subunit and the three β subunits are not identical. The result is three different conformations for the three β subunits: T (tight), L (loose) and O (open). The subunit in T conformation binds ATP very strongly and its affinity for the molecule is so high that it will induce the conversion of ADP + P_i_ into ATP. The subunit in the L conformation, instead, is able to bind ADP and P_i_ but it cannot release the nucleotides. Finally, the O conformation allows the release of the formed ATP. The result of the γ subunit rotation is the change in these subunit conformations allowing the passage through all the stages and the generation of ATP [56]. 

As for the other complexes described, the CV assembly is also modular. Three sub-assemblies are formed individually and then put together: the F_1_ module, the c-ring and the peripheral stalk [395]. The F_1_ subcomplex formation requires the activity of the chaperones ATPAF1/ATP11 and ATPAF2/ATP12, which bind ATP5B and ATP5A1, respectively [396]. Initially, the F_1_ and the c-ring modules assemble. The peripheral stalk, instead, is incorporated in two additional steps: the incorporation of b/ATP5F1, d/ATPH, F_6_/ATP5J and OSCP/ATP5O first and the addition of e/ATP5I, g/ATP5L and f/ATPJ2 in a second step [397,398]. Different assembly factors involved in this process have been identified in yeasts. Atp25 stimulates the synthesis and assembly of subunit c of the c-ring [399], while the protease Atp23 processes and stabilizes the membrane-inserted yeast Atp6, unprocessed in mammals [400]. Finally, the inner membrane assembly complex (INAC), composed of Ina17 and Ina22 [401], binds and stabilizes two distinct assembly intermediates of the yeast ATP synthase: the newly assembled c-ring and an assembly intermediate composed of the F_1_ domain and the peripheral stalk.

#### 2.7.2. Pathologies Associated with Complex V Deficiency

Patients presenting with CV defects are rare and generally associated with neonatal-onset hypotonia and hypertrophic cardiomyopathy, lactic acidosis and 3-methylglutaconic aciduria [193]. Only few pathological mutations in CV subunits or assembly factors have been found in patients so far. The majority of these mutations were identified in MT-ATP6, responsible for neurogenic muscle weakness and ataxia and retinitis pigmentosa (NARP) syndrome [402,403], and MT-ATP8 [404], while rarer cases were found in ATP5E, ATP5A1, ATPAF2 and TMEM70 [404,405,406,407,408,409]. The CV subunits or assembly factors found mutated in patients with CV deficiency and the relative clinical phenotypes are summarized in Table 8. 

### 2.8. Localization of the OXPHOS Machinery in the IMM

The OXPHOS machinery is embedded in the IMM, together with a variety of other mitochondrial proteins. Indeed, the IMM is one of the most protein-rich lipid bilayers in biological systems, with a protein/lipid mass ratio of ~75:25 [415]. Many protein complexes which localize in this compartment are not distributed randomly, but tend to cluster in specific regions, according to their individual function. As discussed previously, the IMM can be divided into two subdomains: the inner boundary membrane (IBM), opposite to the OMM, and the cristae membrane (CM), the alias for invaginations of the membrane in the matrix, connected by the cristae junctions. 

Results obtained with quantitative immunogold-EM on mammalian mitochondria and yeast cells demonstrated that the preferential location of OXPHOS complexes is the CM, but that both subcompartments are dynamic and the distribution of mitochondrial proteins can change according to the physiological state of the cell [416,417]. In particular, it has been observed that OXPHOS enzymes—or intermediates of them—might localize in different regions of the membrane in different stages of maturation. With the only exception of CII, the respiratory chain enzymes are composed of both nuclear- and mitochondrial-encoded subunits, and their biogenesis is the result of the coordination between two temporally and spatially separated protein synthesis machineries. Consequently, it was proposed that, while proteins synthesized in the matrix are translated and inserted directly in the cristae, subunits that must be imported from the cytosol are preferentially inserted in the IBM [417]. More recent analyses by super-resolution microscopy and quantitative cryo-immunogold-EM have helped to determine where specifically CIII, CIV and CV subunits are translated and inserted in the yeast inner membrane [418]. This study confirmed that, under steady-state conditions, the mature forms of CIII, CIV and CV localize mainly in the CM, while early stages of assembly are more enriched in the IBM. Indeed, mitoribosomes translating *COB* mRNA (the very first step of CIII assembly) and Cbp3 and Cbp6 (markers for the early assembly of the enzyme) were found more present in the IBM, while Cbp4 (a marker of the early-to-mid-assembly phase) was already less enriched in this subcompartment. Conversely, the integration of the Rieske protein, the last step of maturation, which takes place after CIII dimerization, happened predominantly in the CM. Similarly, Stoldt and colleagues investigated the localization of markers of the early (Pnt1, Cox18, Coa1 and Cox20) and the late (Pet100) phases of CIV assembly, finding the earliest stages of maturation enriched in the IBM and the late phases in the CM. By contrast, the entire assembly pathway of CV seems to occur mainly in the CM [418].

### 2.9. Supercomplexes

The picture of the OXPHOS machinery as individual enzymes sitting in the IMM is a simplified vision of what happens in the mitochondria of living cells. A second level of complexity is added by the formation of supercomplexes (SC), stable structures composed of the association of the respiratory chain enzymes. 

The development of blue native PAGE techniques [419] allowed the separation and detection of both the individual complexes and the supercomplexes, composed of different combinations of CI, CIII_2_ and CIV. However, many questions remained open in the field. Firstly, it was necessary to characterize the type and strength of these inter-complex interactions, in order to hypothesize a realistic model of IMM organization. Secondly, it was necessary to attempt to explain the biological and physiological function of these structures. This second aspect will be one of the central questions of this thesis. 

#### 2.9.1. Existing Models

The models to explain the organization of the respiratory chain enzymes have changed over time. The first proposal, known as the “fluid state” model, describes the mitochondrial complexes as individual and independent units that float in the inner membrane and collide randomly with each other, allowing the passage of electrons. According to this theory, cytochrome *c* would be diffusing in three dimensions, in order to transport electrons between complexes, and this transient and casual formation of each SC species by collision would allow the cell to adapt to different energy demands. This model was the most accepted one during the 1980s, following the presentation of the random collision model of Hackenbrock [420]. The fluid model is supported by the very high protein-to-lipid ratio in the membrane, which allows the frequent interaction of the enzymes, and by scanning calorimetry [421] and freeze-fracture electron microscopy [422] studies showing that the intramembrane particles are randomly distributed in the IMM. Moreover, independent studies showed that CoQ is a mobile carrier [423,424], confirming an essential part of this theory. On the other hand, however, both blue native PAGE and electron microscopy experiments in different systems, such as mammals, bacteria, yeasts and plants, suggest more stable interactions between these particles [425,426,427,428,429]. In the original experiments, it was necessary to solubilize the membranes with a strong detergent in order to isolate the single enzyme, while milder detergents such as digitonin used in blue native studies preserve the various species of SC intact. 

The second and opposite model proposed was the “solid state” model, which supported the vision that the different activities and redox centers were contained in an undissociated protein matrix. These structures were also thought to contain Q and cyt *c* [430,431]. This view, however, was challenged by the observation that the isolated complexes were functional and that they could diffuse within the lipid bilayer, as well as activity rate values being compatible with a mechanism of random collision [432]. The “solid state” model remained the most accepted view until the development of the native electrophoretic techniques described above, which showed the co-existence of supercomplexes of different sizes and the individual respiratory chain complexes. From a functional point of view, the proximity of the enzymes and the molecular carriers involved would increase the interactions and prevent the intermediates from escaping or being sequestered by other enzymes for use in secondary metabolic pathways [433]. Indeed, in the model in which the different supramolecular species coexist and trap the mobile electron carriers, the association of CI, III and IV and, on the other side, CIII and IV would define two different functional CoQ populations: CoQ dedicated to transferring electrons originating from NADH (CoQ_NADH_), which is trapped in SC containing CI, and free CoQ in the inner mitochondrial membrane for use by CII and other enzymes that use FAD (CoQ_FAD_) [434]. However, this system is not compatible with the kinetics of the CoQ pool, and recent additional studies indicated that cytochrome *c* diffuses freely in the IMS in yeast [433] and that only one CoQ and cytochrome *c* pools are present, accessible to all the enzymes [435]. An additional interesting analysis has been proposed after inserting AOX (an alternative oxidase) in vitro, a cyanide-insensitive quinol oxidase originally from *Trypanosoma brucei*, in bovine heart mitochondria [436]. AOX receives electrons from ubiquinone, bypassing CIII and CIV. In the case of two separate pools of ubiquinone, the “respirasome pool” should continue to provide a substantial flux of electrons through the supercomplex. Instead, a competition between respirasomes and AOX was observed in increasing the AOX supplement, confirming the theory that ubiquinone is not channeled but can move freely in the IMM. 

In conclusion, putting together the information available today, the most realistic proposal is a middle-ground and more dynamic model in which various species of supercomplexes and free respiratory complexes coexist. For now, the model that explains this phenomenon is the so-called “dynamic aggregate” or “plasticity” model, which proposes a dynamic interchange of the complexes in their “free” and associated states into SC in response to varying energetic demands. A premise for the model is that the complexes are pre-assembled individually before associating with each other [437,438]. 

#### 2.9.2. Species of Supercomplexes and Complex–Complex Interactions

Structures of the I_1_III_2_IV and I_1_III_2_ SC from mammals and plants have been resolved by single-particle electron cryo-EM [439,440,441,442] and by electron cryo-tomography (cryo-ET) [443,444]. The yeast *Saccharomyces cerevisiae* lacks CI, and complexes III and IV form two SC species III_2_IV_1_ and III_2_IV_2_, the structures of which have been recently solved [445,446].

In mammals, the main supercomplex species are III_2_IV_1_, I_1_III_2_ and the respirasomes I_1_III_2_IV_1−n_. The existence of a “Megacomplex” with a I_2_III_2_IV_2_ stoichiometry, which might also bind CII, was reported in cultured human cells [441]. Additionally, CIV and CV can form homodimers and CIV is involved in the formation of other high-molecular weight structures not yet identified, but visible in blue native PAGE experiments [395,447,448]. “Megacomplexes” containing also CII have been proposed as well, but their existence is debated. 

**Figure 14:**
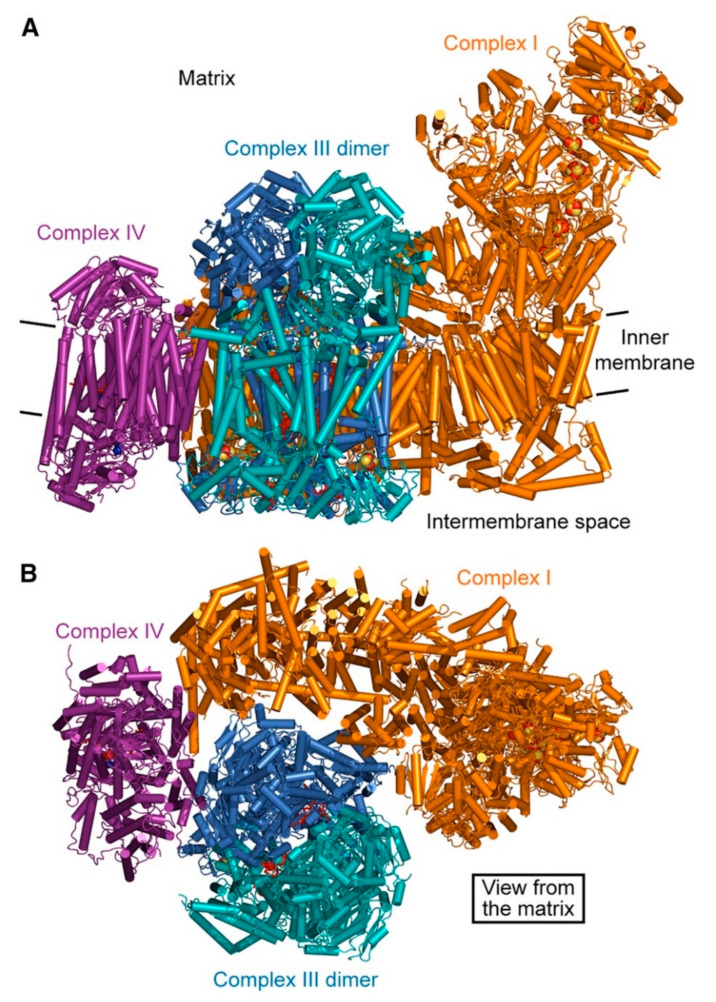
Overall structure of porcine respirasome (I_1_III_2_IV_1_). Source: adapted from Milenkovic et al., 2017 [452].

Since *Saccharomyces cerevisiae* lacks CI [449], yeast-based studies of the respirasomes have been conducted in *Yarrowia* *lipolytica* [444]. In this model, CI and CIII interactions are comparable to what has been observed in mammals, while CIV was found in various positions around CIII_2_, but not in the position most commonly observed in the porcine heart respirasomes, contacting the ND5 module of CI (Figure 14). 

In plants, on the other hand, CIV was never observed in the respirasomes and the only apparent supercomplex was I_1_III_2_. Interestingly, supercomplexes are not universal and simpler organisms such as *Escherichia coli*, which express CI, CII and CV homologues, but not CIII, do not present such structures [450]. In these bacteria, the different complexes have been shown to not co-localize together and to not sub-localize in specific domains over the membrane surface [451]. 

Interestingly, the percentage of free complexes vs. SC varies largely between different species. In bovine heart mitochondria, electron cryo-tomography indicated that only 56% of CI was associated with other complexes [444]. However, BN-PAGE-based quantifications of the same cell type estimated that only 14–16% of free CI was present [426]. On the other hand, blue native-PAGE experiments using human mitochondria show a minimal part of CI (~10% at the most, in the presence of digitonin) migrating as an individual complex [453]. In any case, there seems to be more of “free” CI in mitochondria from different tissues in mammalian organisms such as sheep, pigs or mice than in humans [437,454]. These differences might be due to various reasons, including artefacts introduced during the sample preparation or actual differences in the regulation of energy demands between different organisms. 

In addition, in the same organism, not all the complexes engage in the formation of SC in the same proportion. Indeed, in humans, it has been estimated that while 85%–100% of CI is inserted in a supercomplex structure, only 55%–65% of CIII and 15%–25% of CIV are found in SC [426,453,455,456].

Structural analyses have also been important to define which subunits are involved in the complex–complex interaction. The association between CI and CIV seems to involve subunit COX7A of CIV, and ND5 and NDUFA9 of CI (Letts et al., 2016). COX7A, together with COX8B, could be involved also in the interaction with CIII, through binding of UQCRC1, UQCRQ, UQCR10 or UQCR11 (Letts et al., 2016) [440,444]. Concerning the connection between CI and CIII, subunit NDUFA11 of CI seems to face and contact the transmembrane region of CIII, close to UQCRB and UQCRQ [440] (Letts et al., 2016). Other CI subunits that might be involved in the interaction are NDUFAB1 and NDUFB9, contacting UQCRC1 of CIII. 

#### 2.9.3. Possible Functional Roles of Supercomplexes

The biological function of SC is still highly debated and various hypotheses have been proposed. The first theory proposes that their role is substrate channeling, increasing OXPHOS efficiency due to the physical proximity of all the machinery components [457]. The second possible function of SC might be to minimize ROS production. ROS are generated by the reduction of oxygen by electrons leaked from the ETC and are physiologically used as a signaling molecule [458]. However, if there is an imbalance between the excessive formation of ROS and limited antioxidant defenses, ROS can become deleterious and damage mtDNA, lipids and proteins. In this case, the formation of supra-structures might decrease electron or proton leakages during respiration. This theory is supported by two studies. In the first one, ROS production by CI has been shown to increase in two experimental conditions that inhibit the formation of CI and CIII interactions: treating bovine heart mitochondria or liposome-reconstituted supercomplex I-III with dodecyl maltoside, and reconstructing CI and CIII at a high phospholipid/protein ratio [459]. In the second study, the difference in SC formation in astrocytes and neurons was analyzed, showing how astrocytes, which present a higher percentage of free CI, are characterized by poorer mitochondrial respiration but higher ROS production [460].

The last hypothesis, which will be the basis of the first project presented in this thesis, is that the formation of SC is necessary for the assembly and/or stability of the single enzymes. This theory is supported by the observation that defects in one enzyme can lead to multi-complex deficiencies. Indeed, mutations in MTCYB, fundamental for CIII assembly, also induce CI deficiencies [461,462,463]. Similarly, patients presenting with mutations in CIII assembly factors, such as BCS1L, can display defects in both CI and CIV [464], while the suppression of CIV in mouse fibroblasts affects CI assembly or stability [465]. An explanation for this phenomenon was that CI was destabilized after its complete assembly by an active oxidative stress-triggered degradation [466,467,468]. However, a multistep model for SC assembly that sees CI intermediates binding CIII and CIV subunits before the completion of the mature enzyme was proposed [469]. In the same study, UQCRFS1 was shown to insert preferentially within the III_2_ + IV supercomplex, instead of dimeric CIII_2_. 

Finally, an additional role of SC is reserved to the ATP synthase. CV does not seem to interact with the rest of the respiratory chain, but it is able to form dimers and more complex supra-structures such as tetramers and hexamers [470]. These supercomplexes localize specifically at the bottom of the cristae, where they appear to enforce a strong local curvature on the inner membrane, necessary for the formation of the invagination [471].

#### 2.9.4. Assembly of Supercomplexes

Being that the assembly of the monomeric forms of the OXPHOS enzymes requires the participation of a number of assembly factors, it has been proposed that additional proteins should be involved in the formation of supercomplexes.

The first proposed assembly factor was COX7A2L or SCAF1 (supercomplex assembly factor 1), an isoform of the CIV subunit COX7A, described originally to have a role in the inclusion of CIV in III_2_ + IV and I + III_2_ + IV_1–4_ [434]. However, following studies showed that the lack of COX7A2L affects the formation of the SC III_2_ + IV, but not of respirasomes [453,472,473,474]. Pérez-Pérez data also showed how COX7A2L binds to CIII_2_ early during its assembly, indicating a preferential interaction with this complex [472]. Thus, it might possess a role in the establishment of a checkpoint for the regulation of CIII_2_ levels and its incorporation into SC [453]. 

Another study showed how the incorporation of different COX7A isoforms might determine which CIV-containing species (monomer, dimer or respirasome) is formed [13]. Thus, the CIV subunit COX7A2 is replaced by COX7A2L in supercomplexes with both CIV and CIII, and by COX7A1 in CIV dimers. Moreover, other CIV tissue-specific subunit isoforms might play a role in this, as well as dimers, which appeared to be stabilized by the COX6A2 isoform rather than COX6A1. COX6A subunits are localized at the interface between the two monomers, a position that would favor the regulation of the dimerization. COX7A2 and COX6A1, instead, would favor the free CIV form. 

Analyses conducted in *Saccharomyces cerevisiae* suggested the participation of Rcf1 and Rcf2 in the formation of respirasomes [448]. In yeast, the loss of Rcf1 affects CIV function and the correct insertion of Cox13 (equivalent to human COX6A). These data showed also deficiency in SC formation, but recent reports point to this being an indirect effect due to CIV defects [475,476,477]. While Rcf2 is specific to yeast, two orthologs of Rcf1 have been identified in humans: RCF1a/HIGD1A and RCF1b/HIGD2A. HIGD1A has been confirmed to bind CIV and it has been suggested to affect the interaction between CIV and cytochrome *c*, or to act in a still unclear way on the heme centers [306,307]. Another study found HIGD1A directly involved in CIII biogenesis, promoting the final incorporation of UQCRFS1, and suggested its participation in the formation of CIII-containing SC [478]. Concerning HIGD2A, instead, two independent groups demonstrated that the knockdown of this protein leads to impaired SC formation by the release of CIV from respirasomes [479,480]. However, HIGD2A was identified as a MTCO3 module assembly factor and it is possible that its impact on SC is a pleotropic effect due to impaired CIV biogenesis [478,481].

Concerning oligomerization of CV, the role of post-translational modifications in Atp20 has been investigated [482]. Specifically, the molecular mechanism seems to revolve around the phosphorylation of serine 62, which inhibits the dimerization of the ATP synthase. 

Finally, also the lipid composition of the IMM and, in particular, the level of cardiolipin are important for the stabilization of SC. In yeast lacking Taz1, the acyltransferases involved in the remodeling of cardiolipin and CIII and CIV associations are destabilized [483]. In Barth syndrome patients, harboring mutations in Tafazzin (the human ortholog of Taz1), the assembly and stability of CIV and its SC forms are affected, with a secondary effect also on CI + III associations [484]. 

#### 2.9.5. Conclusive Remarks

Mitochondria fulfil a variety of functions in the cell, including the well-known energy production via oxidative phosphorylation. For this reason, the failure of mitochondria to operate correctly is associated with a wide spectrum of genetic disorders. In this review, we have analyzed, in detail, the structure, the assembly pathway and the organization in supercomplexes of each component of the OXPHOS machinery and how defects in these enzymes are linked to diseases in humans. 

Our review aimed to show the heterogeneity and the complexity behind the still growing group of pathologies normally identified as “mitochondrial diseases”, the pathophysiology of which is still often poorly understood. During the past decade, big advancements in genetic screening and in the study of mitochondrial biology and physiology have been made. However, the diagnosis of mitochondrial disorders is limited to about half of the suspected cases, the genotype–phenotype correlation is not always comprehended and effective therapies are still missing.

Future research will need to continue investigating the biology of this organelle, in order to better understand the molecular and cellular basis of mitochondrial diseases, and identify possible targets for new treatments. 

## Figures and Tables

**Table 2:** CI subunits and assembly factors associated with mitochondrial diseases.

Gene/Protein	OMIM	Associated Phenotype	Reference
*Complex I subunits*			
MTND1	516000	Leber optic atrophy, MELAS syndrome, dystonia, spasticity and myopathy.	[135,136,137]
MTND2	516001	Leber optic atrophy.	[138]
MTND3	516002	Infantile encephalopathy and Leigh syndrome.	[139]
MTND4	516003	Leber optic atrophy and MELAS syndrome.	[140,141]
MTND4L	516004	Leber optic atrophy.	[142]
MTND5	516005	Leber optic atrophy and MELAS syndrome.	[143,144]
MTND6	516006	Leber optic atrophy and MELAS syndrome.	[143,145]
NDUFV1	161015	Severe encephalopathy and neurologic abnormalities.	[146,147]
NDUFV2	600532	Hypertrophic cardiomyopathy, truncal hypotonia and encephalopathy.	[148]
NDUFS1	157655	Growth retardation, axial hypotonia, hepatomegaly, dystonia and persistent hyperlactatemia.	[147]
NDUFS2	602985	Neonatal lactic acidosis and hypertrophic cardiomyopathy.	[149]
NDUFS3	603846	Leigh syndrome, severe axial dystonia with oral and pharyngeal motor dysfunction, dysphagia and a tetraparetic syndrome.	[150]
NDUFS4	602694	Muscular hypotonia, absence of visual and auditive attention and cardiac defects.	[151]
NDUFS6	603848	Fatal infantile lactic acidosis.	[152]
NDUFS7	601825	Leigh syndrome, feeding problems, dysarthria and ataxia.	[153]
NDUFS8	602141	Leigh syndrome, poor feeding and episodes of apnea and cyanosis.	[154]
NDUFA11	612638	Fatal infantile metabolic acidosis, brain atrophy, no motor development and hypertrophic cardiomyopathy.	[155]
NDUFA1	300078	Leigh syndrome, hypotonia, nystagmus, generalized choreoathetosis and decreased reflexes.	[156]
NDUFA2	602137	Leigh syndrome, hypertrophic cardiomyopathy and developmental delay.	[157]
NDUFA6	602138	Intrauterine growth retardation, respiratory insufficiency, lactic acidosis and hypoglycemia.	[158]
NDUFA8	603359	Severe neonatal hypotonia, dysmorphic features, epilepsy and signs of brainstem involvement.	[159]
NDUFA9	603834	Respiratory and metabolic acidosis, hearing loss, apneas and retinitis pigmentosa.	[160]
NDUFA10	603835	Leigh syndrome and delayed psychomotor development.	[161]
NDUFA12	614530	Leigh syndrome, progressive loss of motor abilities, scoliosis and dystonia.	[162]
NDUFA13	609435	Delayed development, hypotonia, poor eye contact, abnormal eye movements, poor feeding, encephalopathy and hearing loss.	[163]
NDUFB3	603839	Encephalopathy, myopathy, hypotonia, developmental delay and lactic acidosis.	[164]
NDUFB8	602140	Leigh syndrome, respiratory failure, seizures, hypotonia, cardiac hypertrophy, failure to thrive and severely delayed psychomotor development.	[165]
NDUFB9	601445	Progressive hypotonia associated with increased serum lactate.	[164]
NDUFB10	603843	Lethal complex I deficiency.	[166]
NDUFB11	300403	X-linked microphthalmia with linear skin defects (MLS) syndrome, cardiomyopathy and other congenital anomalies.	[167,168]
NDUFC2	603845	Leigh syndrome.	[169]
*Complex I assembly factors*			
ACAD9	611103	Cardiorespiratory depression, hypertrophic cardiomyopathy, encephalopathy and severe lactic acidosis.	[114]
FOXRED1	613622	Leigh syndrome, congenital lactic acidosis, athetoid movements of the limbs in early childhood, hypotonia and cerebellar atrophy.	[170]
NDUFAF1	606934	Hypertrophic cardiomyopathy, developmental delay, lactic acidosis, hypotonia and Wolff–Parkinson–White syndrome.	[171]
NDUFAF2	609653	Ataxia, lethargy, nystagmus, hypotonia, optic atrophy and episodic respiratory insufficiency.	[122]
NDUFAF3	612911	Macrocephaly, weak cry, no eye contact, wide anterior fontanel and axial hypotonia.	[123]
NDUFAF4	611776	Severe encephalopathy and antenatal cardiomyopathy.	[124]
NDUFAF5	612360	Facial dysmorphism, progressive lactic acidosis and neurological defects.	[125]
NDUFAF6	612392	Focal seizures, decreased movement and strength, ataxia, lactic acidosis and Leigh syndrome.	[172]
NDUFAF8	618461	Leigh syndrome.	[173]
NUBPL	613621	Infantile-onset hepatopathy, renal tubular acidosis, developmental delay, short stature, leukoencephalopathy, myopathy, nystagmus and ataxia.	[106,107,130]
TIMMDC1	615534	Infantile-onset hypotonia, failure to thrive, delayed or minimal psychomotor development, sensorineural deafness, dysmetria, dyskinetic movements, peripheral neuropathy, nystagmus and Leigh syndrome.	[174]
TMEM126B	615533	Exercise intolerance, muscle weakness, myalgia, early-onset renal tubular acidosis and hypertrophic cardiomyopathy.	[175,176]
COA7	615623	Autosomal recessive spinocerebellar ataxia with axonal neuropathy-3.	[177]

**Table 3:** CII subunits and assembly factors associated with mitochondrial diseases.

Gene/Protein	OMIM	Associated Phenotype	Reference
SDHA	600857	Leigh syndrome, neonatal dilated cardiomyopathy, catecholamine-secreting extra-adrenal paraganglioma.	[194,195,196]
SDHB	185470	Paraganglioma, pheochromocytoma, gastrointestinal stromal tumors.	[197,198]
SDHC	602413	Paraganglioma, gastric stromal sarcoma.	[190,199]
SDHD	602690	Paraganglioma, pheochromocytoma, gastric stromal sarcoma.	[191,199]
SDHAF1	612848	Leukoencephalopathy, spastic quadriplegia, psychomotor regression.	[184]
SDHAF2	613019	Paraganglioma.	[183]

**Table 5:** CIII subunits and assembly factors associated with mitochondrial diseases.

Gene/Protein	OMIM	Associated Phenotype	Reference
Complex III subunits			
UQCRC2	191329	Hypoglycemia, lactic acidosis, ketosis and hyperammonemia.	[274]
MTCYB	516020	Leber optic atrophy, exercise intolerance, encephalomyopathy, cardiomyopathy and multisystemic disorder.	[275,276,277,278,279]
CYC1	123980	Neurologic deterioration, insulin-responsive hyperglycemia, ketoacidosis with increased serum lactate, liver failure and hyperammonemia.	[280]
UQCRB	191330	Gastroenteritis, liver enlargement, hypoglycemia and metabolic acidosis but normal psychomotor development at age 4.	[216]
UQCRQ	612080	Severe neurologic phenotype.	[215]
UQCRFS1	191327	Cardiomyopathy and alopecia totalis.	[281]
Complex III assembly factors			
BCS1L	603647	GRACILE Syndrome, Bjornstad Syndrome, myopathy, encephalopathy, proximal tubulopathy and liver failure.	[272,273,282,283,284,285,286,287,288]
TTC19	613814	Progressive encephalopathy, ataxia, spastic paraparesis and psychiatric phenotype.	[256,289,290,291,292]
LYRM7	615831	Neurological decompensation and regression, leukoencephalopathy and liver failure.	[293,294]
UQCC2	614461	Intrauterine growth retardation, neonatal lactic acidosis and renal tubular dysfunction.	[234,295]
UQCC3	616097	Lactic acidosis, hypoglycemia, hypotonia and delayed development.	[270]

**Table 6:** Factors involved in mammals CIV assembly. When present, the yeast orthologue is indicated.

Assembly Factor (Yeast)	Assembly Factor (Mammals)	Function	CIV Interacting Module	References
RNA stability and translation				
-	TACO1	Translational activator of mitochondria-encoded MTCO1.	MTCO1-translation	[311]
-	LRPPRC	Mitochondrial mRNA stability.	-	[310]
-	FASTKD2	Involved in post-transcriptional RNA maturation, ribosome biogenesis and translation.	-	[333]
Heme a biosynthesis and insertion				
Cox10	COX10	Heme *a* synthesis (conversion of heme *b* into heme *o*).	MTCO1 module	[316,334]
Cox15	COX15	Heme *a* synthesis (conversion of heme *o* into heme *a*).	MTCO1 module	[335,336]
Shy1	SURF1	Involved in the insertion or stabilization of heme *a*_3_.	Early MTCO1 subcomplexes	[337]
Copper metabolism and insertion				
Coa6	COA6	Copper homeostasis and transport to CIV.	MTCO2 module	[328,338]
Sco1	SCO1	Incorporation of copper atoms.	MTCO2 module	[327,339]
-	SCO2	Incorporation of copper atoms.	MTCO2 module	[340]
Cox11	COX11	Copper chaperone.	MTCO1 module	[319,341]
Cox16	COX16	MTCO2 maturation.	MTCO2 module	[331,342]
Cox17	COX17	Copper transfer.	MTCO1 module	[321]
Cox19	COX19	Stabilization of COX11.	MTCO1 module	[320,343]
Assembly				
Coa3	COA3/MITRAC12	Required for MTCO1 stability and assembly. Involved in translational regulation of MTCO1 and prevention of MTCO1 aggregation before assembly.	MTCO1 module	[313,314]
-	COA7	Unknown.	Unknown	[177]
Cox14	COX14/c12orf62	MTCO1 stability and assembly; avoids MTCO1 aggregation.	MTCO1 module	[312,344]
Cmc1	CMC1	Stabilizes the interaction between MTCO1, COX14 and COA3.	MTCO1 module	[315]
-	COX20/FAM36A	MTCO2 chaperone for copper metalation.	MTCO2 module	[345]
Pet100	PET100	Assembly factor.	S3 intermediary	[346,347,348]
Pet117	PET117	Assembly factor; possible role in Cox15 oligomerization and function.	S3 intermediary	[318,349,350]
-	MR-1S	Interacts with PET117 and PET100.	S3 intermediary	[306]
-	APOPT1/COA8	Intermediate assembly steps. Putative role in CIV protection from ROS damage.	Unknown	[351]
Cox18	COX18	Promotes the translocation of MTCO2 globular domain through the IMM.	MTCO2	[352,353]

**Table 7:** CIV subunits and assembly factors associated with mitochondrial diseases.

Gene/Protein	OMIM	Associated Phenotype	Reference
Complex IV subunits			
MTCO1	516030	MELAS syndrome, myopathy, myoglobinuria, motor neurone disease, exercise intolerance, epilepsy, multisystem disorders, deafness, LHON or mitochondrial sensorineural hearing loss.	[355,356,357,358,359]
MTCO2	516040	Encephalomyopathy, LHON, myopathy, hypertrophic cardiomyopathy.	[360,361,362,363]
MTCO3	516050	MIDD, LHON, myopathy, Leigh disease, myoglobinuria, sporadic bilateral optic neuropathy, rhabdomyolysis, encephalopathy.	[364,365,366,367,368,369]
COX4I1	123864	Short stature, poor weight gain, mild dysmorphic features, Fanconi anemia.	[370]
COX4I2	607976	Exocrine pancreatic insufficiency, dyserythropoietic anemia, calvarial hyperostosis.	[371]
COX5A	603773	Early-onset pulmonary arterial hypertension, lactic acidemia, failure to thrive.	[372]
COX6A1	602072	Charcot–Marie–Tooth disease.	[373]
COX6A2	602009	Muscle weakness and hypotonia, cardiomyopathy.	[374]
COX6B1	124089	Severe infantile encephalomyopathy.	[375]
COX7A1	123995	Failure to thrive, encephalopathy, hypotonia.	[376]
COX7B	300885	Microphthalmia with linear skin lesions.	[377]
COX8A	123870	Leigh-like syndrome presenting with leukodystrophy and severe epilepsy.	[378]
NDUFA4	603833	Leigh syndrome.	[298]
Complex IV assembly factors			
SURF1	185620	Leigh syndrome, Charcot–Marie–Tooth disease.	[379,380]
COA3/MITRAC12	614775	Mild phenotype, exercise intolerance, peripheral neuropathy, obesity and short stature.	[308]
COA7	615623	Ataxia and peripheral neuropathy, cognitive impairments, leukodystrophy.	[177]
COX14/c12orf62	614478	Severe lactic acidosis and dysmorphic features.	[344]
COX20/FAM36A	614698	Growth delay, hypotonia, cerebellar ataxia.	[381]
PET100	614770	Early-onset psychomotor delay, seizures, hypotonia, Leigh syndrome.	[347,348]
PET117	614771	Neurodevelopmental regression.	[350]
APOPT1/COA8	616003	Leukodystrophy, neurological signs.	[382]
SCO1/SCO2	603644/604272	Cardioencephalomyopathy, Leigh syndrome-like symptoms, spinal muscular atrophy-like presentations, Charcot–Marie–Tooth disease type 4.	[383,384]
COX10/COX15	602125/603646	Leigh syndrome, encephalopathy, cardiomyopathy, sensorineural deafness and metabolic acidosis.	[316,336]
COA6/C1orf31	614772	Fatal infantile cardioencephalopathy.	[385]
TACO1	612958	Leigh syndrome.	[311]
COA5	613920	Fatal infantile cardioencephalomyopathy.	[386]
FASTKD2	612322	Brain atrophy, epilepsy, delayed psychomotor development, bilateral optic atrophy, spastic hemiparesis, cardiomyopathy.	[387,388]
LRPPRC	607544	French Canadian type of Leigh syndrome.	[389]

**Table 8:** CV subunits and assembly factors associated with mitochondrial diseases.

Gene/Protein	OMIM	Associated Phenotype	Reference
MT-ATP6	516060	Neuropathy, ataxia and retinitis pigmentosa (NARP), maternally inherited Leigh’s syndrome (MILS), mental retardation, ataxia, cardiomyopathy.	[402,403,410,411]
MT-ATP8	516070	Hypertrophic cardiomyopathy and neuropathy.	[404]
ATP5E	614053	Neonatal-onset lactic acidosis, 3-methylglutaconic aciduria, mental retardation, hypertrophic cardiomyopathy and peripheral neuropathy.	[405]
ATP5A1	615228	Fatal infantile encephalopathy.	[408]
ATPAF2	608918	Degenerative encephalopathy, elevated lactate levels, developmental delay.	[412]
ATP5F1A	164360	Fatal infantile mitochondrial encephalopathy	[408,413]
ATP5F1D	603150	Metabolic decompensation with lactic acidosis, hypoglycemia, hyperammonemia, 3-methylglutaconic aciduria, encephalopathy.	[414]
ATP5F1E	606153	Neonatal-onset lactic acidosis, 3-methylglutaconic aciduria, mild mental retardation, hypertrophic cardiomyopathy, peripheral neuropathy.	[405]
TMEM70	612418	Neonatal mitochondrial encephalocardiomyopathy.	[119]

## Data Availability

The data reported in this review are taken from publicly available, peer-reviewed scientific papers.
